# Bias in mobility datasets drives divergence in modeled outbreak dynamics

**DOI:** 10.1038/s43856-024-00714-5

**Published:** 2025-01-07

**Authors:** Taylor Chin, Michael A. Johansson, Anir Chowdhury, Shayan Chowdhury, Kawsar Hosan, Md Tanvir Quader, Caroline O. Buckee, Ayesha S. Mahmud

**Affiliations:** 1 Center for Communicable Disease Dynamics, Department of Epidemiology, Harvard T.H. Chan School of Public Health, Boston, MA USA; 2 Bouvé College of Health Sciences & Network Science Institute, Northeastern University, MA Boston, USA; 3 a2i, Dhaka, Bangladesh; 4 Department of Psychiatry, Columbia University Irving Medical Center, New York, NY USA; 5 Department of Economics, Jahangirnagar University, Dhaka, Bangladesh; 6 Department of Demography, University of California, Berkeley, California, USA

**Keywords:** Infectious diseases, Respiratory tract diseases

## Abstract

**Background:**

Digital data sources such as mobile phone call detail records (CDRs) are increasingly being used to estimate population mobility fluxes and to predict the spatiotemporal dynamics of infectious disease outbreaks. Differences in mobile phone operators’ geographic coverage, however, may result in biased mobility estimates.

**Methods:**

We leverage a unique dataset consisting of CDRs from three mobile phone operators in Bangladesh and digital trace data from Meta’s Data for Good program to compare mobility patterns across these sources. We use a metapopulation model to compare the sources’ effects on simulated outbreak trajectories, and compare results with a benchmark model with data from all three operators, representing around 100 million subscribers across the country.

**Results:**

We show that mobility sources can vary significantly in their coverage of travel routes and geographic mobility patterns. Differences in projected outbreak dynamics are more pronounced at finer spatial scales, especially if the outbreak is seeded in smaller and/or geographically isolated regions. In some instances, a simple diffusion (gravity) model was better able to capture the timing and spatial spread of the outbreak compared to the sparser mobility sources.

**Conclusions:**

Our results highlight the potential biases in predicted outbreak dynamics from a metapopulation model parameterized with non-population representative data, and the limits to the generalizability of models built on these types of novel human behavioral data.

## Abstract (web summary)

Chin et al. use a metapopulation model to compare mobile phone movement data’s effects on simulated outbreak trajectories. Potential biases in predicted infectious disease outbreak dynamics are seen in a metapopulation model parameterized with human behavioral data from mobile phone and social media users.

## Plain Language Reporting Summary

Movement of people can be estimated from mobile phone location tracking data. These data are increasingly being used to predict the spread of infectious diseases. We built mathematical models using different sources of mobile phone location tracking data to show the potential biases, and the limits to the generalizability, of infectious disease models built on these types of novel human behavioral data. We found that different sources of location data varied substantially in their spatial coverage. These differences can impact the ability to use these sources of information to predict the spread of infectious diseases.

## Introduction

Spatiotemporal patterns of human mobility and aggregation have been shown to be influential in driving the spatial spread of outbreaks for a variety of pathogens^1–8^. Access to accurate and timely data on human mobility is therefore critical for responding effectively to epidemics. Digital data sources like call detail records (CDRs), which are attributes of calls and SMS-texts routinely collected by mobile phone operators for administrative purposes, are increasingly being used to understand mobility patterns in relation to infectious disease transmission dynamics. Use of mobile phone data for modeling research during the SARS-CoV-2 pandemic, in particular, has increased awareness of these digital data sources, resulting in efforts to standardize definitions of mobility metrics and workflows^9,10^.

CDRs, which capture data from any individual who has a phone with a SIM card used for calling and messaging, are particularly valuable as a source of mobility data in resource-poor countries, where many individuals still do not have smartphones. CDR-derived mobility estimates have been shown to outperform more traditional mobility estimate sources, including census commuter data and gravity-type mobility networks, in reproducing local outbreak dynamics^11,12^. From a practical perspective, since CDRs are routinely collected by telecom operators for administrative purposes, they represent a relatively low-cost and efficient data source for researchers to use, provided data sharing arrangements can be established between researchers and the mobile phone operators. For these reasons, CDR-derived mobility estimates have been used to model the transmission dynamics of various infectious diseases, including rubella^4^, malaria^13,14^, dengue^15,16^, Zika^12^, cholera^1,17^, and SARS-CoV-2 ^18^.

Despite some of the advantages of CDR data, potential bias in mobile phone ownership is a well-established challenge when interpreting mobility estimates from mobile phone data. Mobile phone owners in Kenya and Rwanda, for example, were found to be more likely to be males with higher income and education and from larger households relative to people without mobile phones^19,20^. At the same time, however, other work has suggested that mobility estimates based on CDR data may be relatively robust to these demographic differences in ownership^21^.

While previous work has illuminated both the promise and some limitations of CDR data^22–24^, the impact of biased geographic representativeness has hitherto been underexplored. This bias arises due to differences in geographic coverage of mobile phone operators based on the location of their subscriber base, their subscribers’ travel-relevant characteristics (e.g. socioeconomic status, work travel demands) that influence their operator choice, and the location of their cellphone towers. For example, an operator might lack cell phone towers in certain regions of a country and therefore fail to capture mobility patterns in these regions, whereas other operators might have towers in the same regions. Using CDR data from one mobile phone operator is a common practice in research due to data access constraints. Reliance on data from a sole operator, however, may introduce unclear biases based on that operator’s unique geographic coverage area. The extent to which modeling conclusions may change depending on access to CDR data from different mobile phone operators remains poorly understood and quantified.

Here, we addressed these gaps by leveraging datasets from three (of four) mobile phone operators in Bangladesh. We analyzed differences in CDR-derived mobility patterns across these three operators and also compared these estimates to mobility estimates derived from digital trace data from Meta’s Data for Good program, which is based on data from smartphone users. We then used a metapopulation model with spatial coupling between geographic units parameterized via these mobility estimates to simulate the outbreak of a hypothetical respiratory pathogen seeded in different cities in Bangladesh. We compared simulations with a benchmark model parameterized with data from all three operators, to evaluate each of the mobility sources’ effects on simulated outbreak trajectories. In the absence of high-resolution population-representative mobility data, the benchmark model provides the closest approximation available for “ground-truth” data, with the combined CDR data representing 100 million subscribers out of the total country’s population size of 169 million people. We find that mobility sources can vary significantly in their coverage of travel routes and geographic mobility patterns. The differences in the mobility sources had substantial impact on the simulated epidemics, including whether the epidemics spread across the country, how fast they spread, the geographic pattern of spread, and the overall impact. Our results show that differences in projected outbreak dynamics are more pronounced at finer spatial scales, especially if the outbreak is seeded in smaller and/or geographically isolated regions. In some instances, a simple diffusion (gravity) model was better able to capture the timing and spatial spread of the outbreak compared to the sparser mobility sources.

## Methods

### Call detail record data

We derived mobility estimates from Call Detail Records (CDR) data obtained from three mobile phone operators in Bangladesh using methods previously described^3,4,15,25^ and summarized here. In brief, subscribers were assigned to an upazila—one of 544 sub-district administrative units—each day in the analysis period, April 28, 2020 to September 1, 2020, based on their most frequently used cell tower for routing calls and SMS texts. Trips between upazilas were defined as a change in a subscriber’s assigned tower location compared to the previous day. The aggregated data received from each operator consisted of the daily number of trips taken between all pairs of upazilas and the number remaining in each upazila from one day to the next.

Due to our privacy sharing arrangements with the operators, we refer to them only as Operators 1, 2, and 3. Together these three operators represent approximately 100 million subscriptions in Bangladesh, which has a total population size of approximately 169 million. To ensure anonymity, the mobile phone data were aggregated temporally at a daily scale as well as spatially at the upazila administrative level. All three operators generated this data for public health use during the first year of the COVID-19 pandemic. The operators used the same methods for generating the aggregated data; however, there might still be differences across operators in how the original raw CDR data was generated and stored.

Although the data were from 2020 during the COVID-19 pandemic, which disrupted absolute travel volumes, we assumed the travel patterns in terms of the proportions of people traveling between pairs of upazilas to be representative of normal travel patterns. To check the validity of this assumption, we used data from Operator 1 from 2017 (*i.e*., data prior to the COVID-19 pandemic), which is the only operator with available data from 2017, to estimate the proportions of people traveling between pairs of upazilas on weekdays. We averaged over weekdays because of differences in the date ranges with available data for 2017 and 2020. The 2017 data from Operator 1 consists of approximately 64 million subscribers and covers the time period from April 1-September 30, 2017. The correlation between upazila-to-upazila mobility proportions from 2017 and those from 2020 was estimated as r = 0.73 (Supplementary Fig. 1. Based on this high correlation between 2017 and 2020 values, we made the assumption that data from 2020 may be representative of relative travel patterns (e.g., proportion of travelers traveling from Dhaka to all other upazilas in these time periods) despite disruptions to the absolute number of people traveling in 2020. To generate mobility estimates that are representative of typical travel patterns, we removed the dates May 24-May 26, 2020 and July 31-August 2, 2020 from the datasets since these dates represent the Eid holidays, which disrupt average travel patterns.

### Meta Data for Good

In addition to CDR data, we analyzed mobility estimates from Meta’s Data for Good program^26^. Since Meta’s Data for Good is free and openly available to response workers and researchers who request access, it has been used for mobility estimates in several modeling studies, especially during the SARS-CoV-2 pandemic^27–29^. While data from Meta is more accessible to researchers than CDR data, there are important differences in the populations captured by the different data sources. CDR users include people who have a cell phone with a SIM card, whereas Meta’s Data For Good captures an open cohort of people who meet the following criteria: (1) have a smartphone, (2) have the Meta application on their phone, and (3) have shared their location history data by having location services enabled on their phone.

We used Meta’s Movement Between Tiles and Population (Tile Level) datasets^30^ from the time period March 21-September 30, 2020. User location is categorized by Bing Tiles. Population data are based on users’ most frequently visited tile during 8-hour windows. The Movement Between Tiles data consist of movement vectors estimated based on changes in users’ modal location in the current 8-hour window compared to their modal location in the preceding 8-hour window. Lastly, to help preserve anonymity, if the number of users is below 10 users for a given 8-hour window, the observation is dropped from the dataset.

We calculated district-level values by mapping the Bing Tiles’ centroids to districts and summing across values that fell in each district. Daily estimates were calculated by taking an average across the three, 8-hour windows per day.

### Gravity model

To compare mobility estimates from CDR and Meta, we compared against two null gravity models constructed at the district and upazila levels. We calculated the number of people traveling from location *j* to location *i*, ${X}_{{ij}}$, using a gravity model as:

1 $${X}_{{ij}}=k({p}_{i}^{\alpha }* {p}_{j}^{\beta })/{d}_{{ij}}^{\gamma }$$

Where *k* is a constant, ${p}_{i}$ and ${p}_{j}$ are the populations of location *i* and location *j*, respectively, ${d}_{{ij}}$, is the Euclidean distance between location *i* and location *j*, and $\alpha ,\beta ,\gamma$ are parameter estimates from the literature^31^. In the main results, we present simulation results using a gravity model with parameters from existing literature, rather than fitting a gravity model fit to the CDR data from Bangladesh, as we believe this is a better representation of what a researcher or practitioner might use in the absence of other sources of data. As a comparison, we also fit the gravity model to the combined data from Operators 1, 2, and 3. We fit a negative binomial regression model to the observed count of trips between locations with ${\log p}_{i}$, ${\log p}_{j}$, and ${\log d}_{{ij}}$ as the independent variables (results are in Supplementary Table 1). We use the fitted parameters from the regression model to estimate ${X}_{{ij}}$. The gravity model with parameters from literature and the fitted gravity model are remarkably similar, and produce similar deviations in simulated disease dynamics compared to the benchmark model (Supplementary Fig. 2, Fig. 3).

### Descriptive data sources

Upazila- and district-level shapefiles are from The Humanitarian Data Exchange^32^. Upazila-level population estimates from 2020 are from WorldPop^33^. We summed across rasters with centroids that fell in each upazila. We then summed across upazilas to get district-level population estimates. Average annual nightlight levels per district from 2020 were used as a proxy for the degree of urbanicity and obtained from the Earth Observation Group – VIIRS Nighttime Lights dataset^34^. Mean household income in USD for each union, which is a sub-upazila administrative unit, from 2013 is from WorldPop^35^. We took an average of values across unions and upazilas to get upazila-level and district-level estimates, respectively.

### Metapopulation model

We simulated the outbreak of a hypothetical emerging respiratory pathogen using a metapopulation model structure to incorporate spatial coupling between upazilas. The model was adapted from Li et al*.*^36^, and can be summarized by the following equations:

2 $$\frac{d{S}_{i}}{{dt}}=-\frac{\beta {S}_{i}{{I}_{i}}^{S}}{{N}_{i}}-\frac{\alpha \beta {S}_{i}{{I}_{i}}^{A}}{{N}_{i}}+{\sum }_{j}\frac{{M}_{{ij}}{S}_{j}}{{N}_{j-}{{I}_{j}}^{S}}-{\sum }_{j}\frac{{M}_{{ji}}{S}_{i}}{{N}_{i-}{{I}_{i}}^{S}}$$

3 $$\frac{d{E}_{i}}{{dt}}=\frac{\beta {S}_{i}{{I}_{i}}^{S}}{{N}_{i}}+\frac{\alpha \beta {S}_{i}{{I}_{i}}^{A}}{{N}_{i}}-{\sigma E}_{i}+{\sum }_{j}\frac{{M}_{{ij}}{E}_{j}}{{N}_{j-}{{I}_{j}}^{S}}-{\sum }_{j}\frac{{M}_{{ji}}{E}_{i}}{{N}_{i-}{{I}_{i}}^{S}}$$

4 $$\frac{d{{I}_{i}}^{S}}{{dt}}=\delta {\sigma E}_{i}-\gamma {{I}_{i}}^{S}$$

5 $$\frac{d{{I}_{i}}^{A}}{{dt}}=\left(1-\delta \right){\sigma E}_{i}-\gamma {{I}_{i}}^{A}+{\sum }_{j}\frac{{M}_{{ij}}{{I}_{i}}^{A}}{{N}_{j-}{{I}_{j}}^{S}}-{\sum }_{j}\frac{{M}_{{ji}}{{I}_{i}}^{A}}{{N}_{i-}{{I}_{i}}^{S}}$$

6 $${N}_{i}={N}_{i}+{\sum }_{j}{M}_{{ij}}-{\sum }_{j}{M}_{{ji}}$$

where${S}_{i},{E}_{i},{{I}_{i}}^{S}$, ${{I}_{i}}^{A},{N}_{i}$ are the susceptible, exposed, symptomatic infectious, asymptomatic infectious, and total population in upazila $i$, respectively; $\beta$ is the transmission rate of symptomatic, infectious individuals; $\alpha$ is the factor by which the transmission rate is multiplied for asymptomatic infectious individuals; $\delta$ is the probability of having clinical symptoms; $\sigma$ is the inverse of the latent period; $\gamma$ is the recovery rate; and ${M}_{{ij}}$ is the daily average number of subscribers traveling from upazila *j* to upazila *i*

As in Li *et al*., we assumed that symptomatic infectious individuals do not travel, although they can travel during the latent period^36^. The model was integrated stochastically using a fourth order Runge-Kutta (RK4) scheme, where for each step of the RK4 scheme, each term on the right-hand side of Eqs. (2)-(5) was based on a random sample drawn from a Poisson distribution^36^.

Given the analysis’ focus on understanding the impact of geographic bias in mobility estimates rather than simulating an actual outbreak, we fixed some of the assumptions for natural history parameters (Table 1). The parameter values were chosen not to represent any specific disease, but rather to model an outbreak of an emerging respiratory pathogen with epidemiological parameters that are in the range of observed parameters for respiratory pathogens in human populations. However, we incorporated variability in the transmission coefficient, $\beta ,$ by using four values for the basic reproduction values, ${R}_{0}$, because of its importance in outbreak dynamics. The basic reproduction number, ${R}_{0}$, and the transmission coefficient are related through the expression for ${R}_{0}$, which was computed as the dominant eigenvalue of the next-generation matrix as the following:

7 $${R}_{0}=\frac{\beta }{\gamma }[\delta +(1-\delta )\alpha ]$$

**Table 1:** Parameter definitions and value assumptions for the metapopulation model

Parameter	Definition	Value
${R}_{0}$	Basic reproduction number	(1.2, 1.3, 1.5, 2)
$\beta$	Transmission coefficient for symptomatic infectious individuals	Calculated based on assumptions for ${R}_{0}$ and expression for ${R}_{0}$
$\alpha$	Multiplicative factor by which transmission coefficient is multiplied for asymptomatic vs. symptomatic infectious individuals	0.5
$\delta$	Proportion of infections that are symptomatic	0.65
$1/\sigma$	Mean latent period	3 days
$1/\gamma$	Mean infectiousness period	5 days

While we intentionally do not model a specific disease, we chose a range of$\,{R}_{0}$ values that are close to empirical estimates for influenza and COVID-19 across a wide range of settings. Varying ${R}_{0}$ impacts the final size of the outbreak, the peak incidence, the timing of the peak, and spatial spread of the disease. With higher transmission rates (higher$\,{R}_{0}$) the pathogen spreads more quickly both within and across districts leading to an earlier and more intense outbreak peak (Supplementary Fig. 4). We ran three sets of simulations based on three different seed cities in Bangladesh – Dhaka, Chittagong, and Panchagarh. These cities were chosen because of their range in population sizes and geographic locations, which were varied to test the impact of different seed conditions on outbreak trajectories: Dhaka and Chittagong represent large urban cities in the center and southern most region of Bangladesh, respectively, while Panchagarh represents a less populous and more geographically isolated area in the north. The three locations were chosen to illustrate the impact of the outbreak starting in highly connected and densely populated locations (Dhaka, followed by Chittagong) versus a smaller, more rural area (Panchagarh). Dhaka and Chittagong were also chosen as they are the largest cities in the country with high connectivity both domestically and internationally. In previous outbreaks, both of these locations have shown to be important for emergence and spread of pathogens^1–3^. With four different value assumptions for the basic reproduction number and three different seed cities, 12 sets of simulations were run at both the district and upazila levels. The CDR and Meta data were used to parameterize the rates at which individuals move between pairs of upazila, ${M}_{{ij}}$, the calculations for which are described later.

Due to the geographic resolution of the tiles in Meta’s data, the highest resolution spatial scale at which we could derive mobility estimates using Meta’s Data For Good was the district level. District-level simulations therefore used population-level mobility estimates from Meta, the three CDR operators, and a district-level gravity model. Upazila-level simulations used mobility estimates from the three CDR operators and an upazila-level gravity model. To allow for comparisons across Meta and CDR data, we present the main findings in terms of the results of district-level simulations. We also discuss findings from upazila-level simulations and include their results in the Supplementary Information. District-level simulations were initialized with 500 people in the latent compartment in the seeded district. As a sensitivity analysis, we also initialized the district-level simulations with 200 and 1000 people in the latent compartment (Supplementary Fig. 5**)**. Changing the initial number infected changed the timing of the outbreak peak but did not substantially alter our results. Upazila-level simulations, meanwhile, were initialized with 100 people in each of the five most populous upazilas in the seeded district (Supplementary Table 2).

We ran each set of district-level simulations 1000 times and quantified 95% uncertainty bounds for the number of people in each of the compartments across the simulation runs. Upazila-level simulations were run 100 times to limit computational demands. Differences in outbreak trajectories across the runs capture uncertainty from two sources: (1) the stochastic Poisson processes in the transmission model and (2) sampling from mobility matrices, which is described later. All simulations were run for 75 weeks (525 days). Incidence shown in the main results figures was calculated as the number of symptomatic infectious individuals, as asymptomatic individuals are likely not captured by surveillance.

### Construction of origin-destination mobility matrices for the metapopulation model

We transformed the aggregated raw CDR data received from the operators into origin-destination mobility matrices using the following steps. For each operator, the aggregated raw data describe the daily number of trips taken between pairs of upazilas and the number of subscribers who stay put in each upazila. Since the three operators have different date coverages in the time period April 28 to September 1, 2020 (excluding the Eid holidays), we calculated average weekend day values and average weekday values across the time period for each data source (Supplementary Fig. 6**)**. We defined the aggregated mobility matrices for each operator as **Q** with elements$\,{q}_{{ij}}$ at row *i* and column *j*, which represents the average daily number of subscribers traveling from upazila *j* to upazila *i*, with missing values imputed, as described in the Supplementary Information. When upazila *i* to upazila *j* reference the same upazila, the matrix element represents the average number of subscribers who start and remain in that same upazila on a given day.

We incorporated uncertainty from the data sources by accounting for differences in their number of subscribers. For each mobility matrix element, ${q}_{{ij}}$, we estimated a beta distribution using Bayesian inference (*binom bayes* function in the *binom* R package), which uses a naïve beta prior (alpha=0.5, beta=0.5) on the probability of success for a binomial distribution to estimate a beta posterior, such that upazilas with few subscribers will have high uncertainty. Here, the number of successes was the matrix cell value, representing the number of people traveling between two upazilas, and the number of independent trials was the matrix column total, which represents the number of people in each upazila who stay put or travel from that upazila in a given day, a proxy for the number of subscribers in the upazila. To populate a simulated mobility matrix with uncertainty incorporated, **B**, we drew a random value for ${b}_{{ij}}$ from the beta distribution defined by the alpha_i,j_ and beta_i,j_ shape parameters estimated for each mobility matrix cell value.

We then normalized the resulting matrix to create matrix **H** with elements ${h}_{{ij}}$ by calculating the proportion of each cell value to its respective column total:

$${h}_{{ij}}={b}_{{ij}}/{\sum }_{i=1}^{n}{b}_{{ij}}$$

where *n* is the number of upazilas. Each ${h}_{{ij}}$ is therefore a realization of a proportion of all subscribers who are residents of *i* that move to each *j* on an average day. We assumed that these proportions for subscribers are equivalent to the proportions of the entire population and multiplied **H** by a diagonal matrix:

$${{{\boldsymbol{K}}}}={{{\boldsymbol{H}}}}* {diag}({{{\boldsymbol{p}}}})$$

where ***p*** is a vector of upazila population sizes (*i.e*. ${{{\boldsymbol{p}}}}={[{p}_{1,}\ldots {,p}_{n}]}^{T}$ for *n* upazilas). Matrix **K** represents the average daily number of people in the population traveling from upazila *j* to upazila *i*.

Lastly, we calculated a symmetric matrix **S** by taking the average of reciprocal values in **K** to balance upazila-level population inflows and outflows:

$${{{\boldsymbol{S}}}}=({{{\boldsymbol{K}}}}+{{{{\boldsymbol{K}}}}}^{T})/2$$

where ${{{{\boldsymbol{K}}}}}^{{{{\boldsymbol{T}}}}}$ is the transpose of matrix **K**. For district-level simulations, we summed upazila values in **S** based on their districts to create a corresponding district-level mobility matrix, **D**. The final origin-destination mobility matrices, **M**, used as inputs in the metapopulation model are either **S** in the case of the upazila-level simulations or **D** for district-level simulations (Supplementary Table 3). To parameterize the mobility matrix in the metapopulation model, we created a weekly vector by repeating the weekday mobility matrix five times followed by repeating the weekend mobility matrix twice, and ran simulations for 75 weeks.

The metapopulation model was initialized using a population vector containing the sums of the mobility matrix columns, representing the effective population sizes of each upazila or district. Conceptually, if an operator’s data indicates more travel to a specific upazila compared to travel from that upazila, the effective population size for that upazila will be larger than its actual population size. This type of upazila would represent one that receives a lot of visitors in a given day. While the effective population size for Bangladesh is the same as the country’s actual population size, the effective population size accounting for mobility and actual population size diverge for districts and especially for less populous districts (Supplementary Fig. 7).

**Fig. 1:**
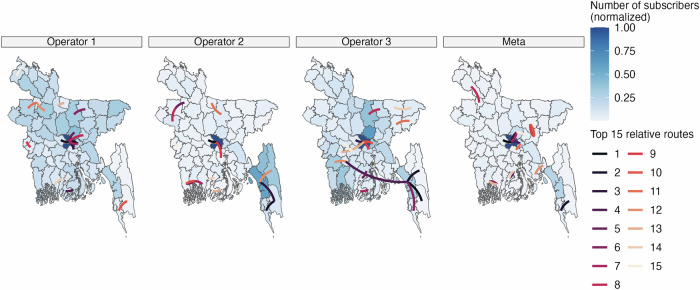
Number of subscribers and top travel routes for each mobility data source. The district color represents the normalized number of subscribers across districts for each mobility data source, defined as the number of subscribers in each district divided by the number of subscribers in Dhaka.The line color represents the top 15 routes by relative volume from each mobility data source, where routes are ranked by the estimated number of travelers between districts divided by the population of the origin district.

For the benchmark model, we used the combined data from Operators 1, 2, and 3 to create the mobility matrix. Together these three operators represent approximately 100 million subscriptions in Bangladesh, which has a total population size of approximately 169 million. While some mobile phone users in Bangladesh may have multiple mobile phones and SIM cards from different operators, leading to potential double counting in the data, this is likely to only be a small fraction of the population given the distinct geographic coverage pattern for each operator (Fig. 1). We were unable to find other population-representative data sources that can provide the spatial and temporal resolution needed to compare with our data sources and to model disease dynamics. We did not include the data from Meta for two main reasons: 1) the Meta data is digital trace data from users with smartphones, thus ensuring overlap with the CDR data; 2) the Meta data is also derived using a different algorithm and it was not possible to obtain upazila-level estimates.

All analyses were conducted in R version 4.2.1. The analyses presented here involves the secondary analysis of existing data that has been de-identified and aggregated. No identifiable private information was available to authors, and this research does not fall within the regulatory definition of research involving human subjects.

### Reporting summary

Further information on research design is available in the Nature Portfolio Reporting Summary linked to this article.

## Results

### Geographic Differences in Coverage

The mobile phone operators differed in terms of their geographic representativeness based on the normalized number of subscribers across the 64 districts (Fig. 1). Dhaka, the capital in the center of Bangladesh, had the highest normalized number of subscribers relative to all other districts for all three operators. Operator 1’s coverage was geographically dispersed with coverage in most districts, Operator 2 had concentrated coverage in the southern Chittagong Hill Tracts region, and Operator 3 had higher geographic coverage in the northern Mymensingh division and southwestern Khulna division. Meta’s coverage was highly concentrated in Dhaka. The four different mobility sources also showed different patterns for the most frequent travel routes, defined as the number of trips divided by the origin district population size (Fig. 1). Several of Operator 1’s top routes were centered around travel to and from Dhaka. The top routes for Operators 2 and 3, meanwhile, were to and from the southern Chittagong Hill Tract districts. Meta’s top routes were distributed throughout Bangladesh.

The pronounced differences in coverage between Meta versus the three mobile phone operators reflect the demographic differences between Meta users and mobile phone subscribers. Since smartphones are not widely adopted yet in Bangladesh, districts with many Meta users were, on average, districts with higher household income and urbanicity levels (Supplementary Fig. 8). By contrast, the number of Operator 1 subscribers across districts was more weakly correlated with districts’ mean household income and urbanicity levels.

**Fig. 2:**
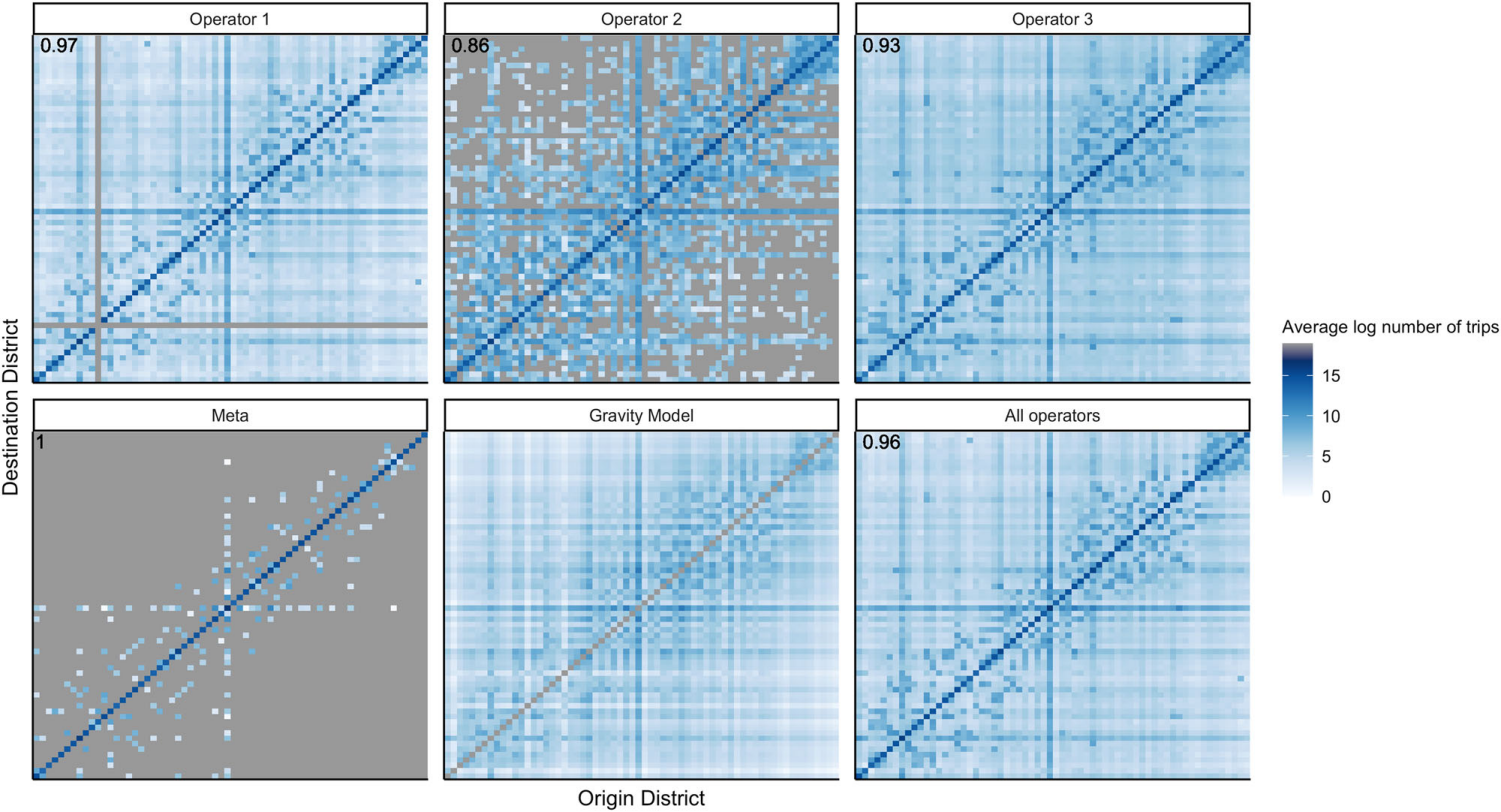
District-level origin-destination matrices. The values reflect the daily average log number of trips taken by people in the population on weekdays in 2020 after excluding the Eid holidays. The matrices have been made symmetric, and districts have been ordered by latitude. The proportion of trips taken within a district vs. to other districts, averaged across all districts, is shown in the top left corner. The gravity model is only used to predict travel between districts, and there are no predictions on within-district travel (along the diagonal).

The upazila-level origin-destination matrices reveal substantial differences across operators in the number of upazila pairs with travel (Supplementary Fig. 9A). Operator 1’s data included trips between upazilas for approximately 93% of all upazila pairs, whereas Operator 2’s matrix was the sparsest, with only approximately 10% of upazila pairs having trips between them and 85% of all pairwise upazilas with no recorded travel between them. Operator 3 fell in between Operators 1 and 2 in terms of matrix sparsity, with 78% of upazila pairs having trips between them. These differences in matrix sparsity on the upazila-level are reflected at the district level (Fig. 2). Meta’s district-level matrix was the sparsest of the four data sources, with several district pairs that have no travel between them.

The operators also differed in terms of the proportion of their subscribers who move every day, which is reflected most clearly in the proportion who do not move (Fig. 2, Supplementary Fig. 9B). Operator 1’s data had higher proportions of people staying put across its districts (mean = 0.97; IQR = 0.01), contrasting Operator 2’s lower and more heterogeneous proportions (mean = 0.86; IQR = 0.06). Operator 3 fell between Operator 1 and 2 (mean = 0.93; IQR = 0.02). Meta’s data had the highest proportion of people staying put in their origin district (mean = 0.99; IQR 0.004) (Fig. 2). On average, more people therefore traveled between upazilas based on Operator 2’s data compared to data from Operator 1 and 3. Of note, although Operator 2’s matrix was sparser than Operator 1 or 3’s, the upazila pairs with no connectivity in Operator 2 data were upazilas with smaller populations on average and also had low connectivity in data from Operators 1 and 3 (Supplementary Fig. 10). In summary, Operator 2 data had higher overall mobility for subscribers but captured no travel for some locations, which had low levels of travel in the other Operator datasets.

### Outbreak Trajectory Differences

**Fig. 3:**
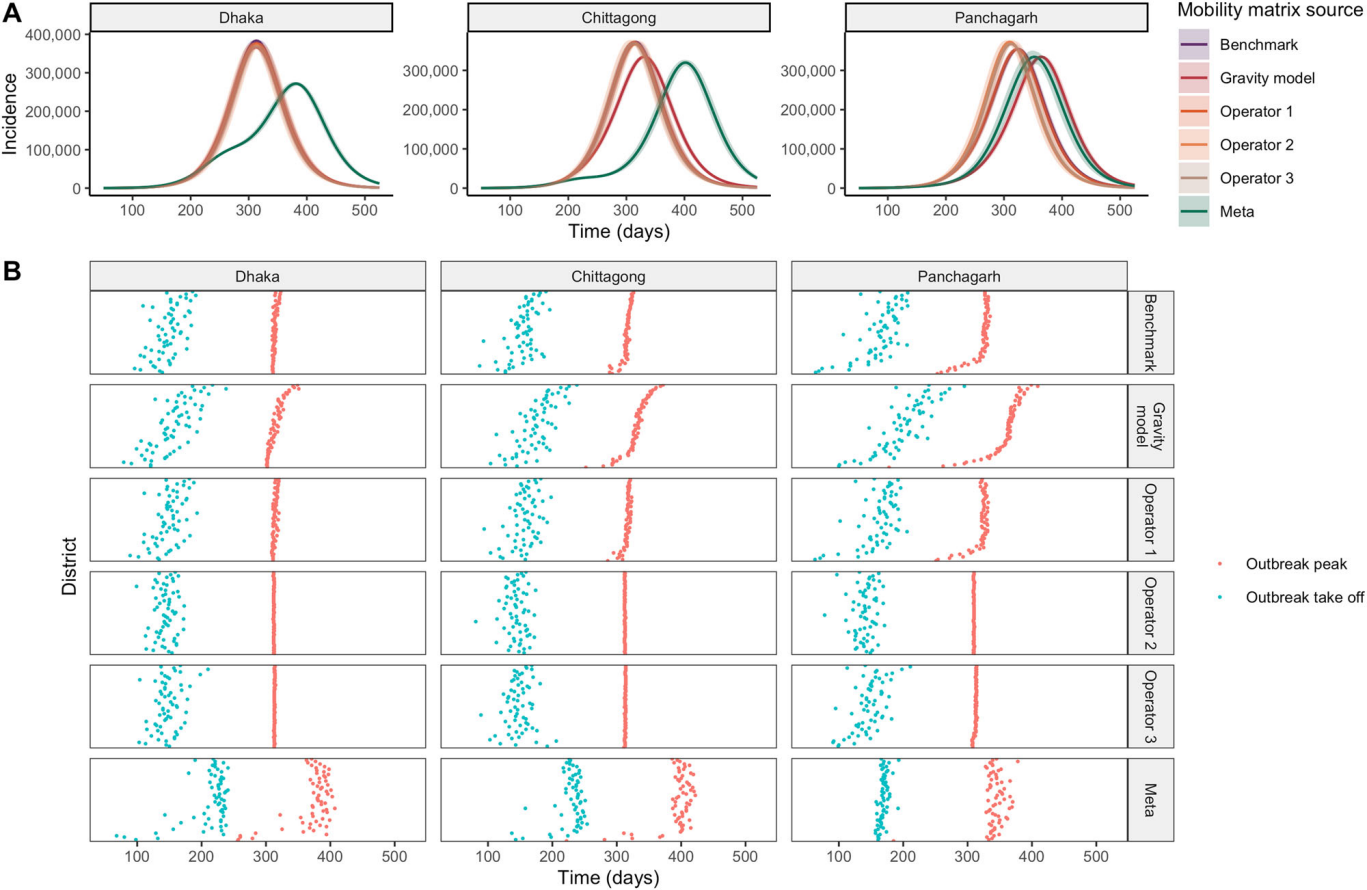
Outbreak dynamics by seed city and mobility data source. Incidence of symptomatic, infectious individuals for all of Bangladesh over time by seed city and mobility matrix source when ${R}_{0}=1.3$ (**A**). Time the outbreak peaks and time the outbreak takes off ($\ge$50 cumulative symptomatic infected cases) across districts by seed city and mobility matrix source. The district order on the y-axis is ordered by distance to the seed city, with the closest district to the seed city on the bottom and the farthest district on the top of each plot (**B**).

The three operators and the gravity model predicted similar country-wide incidence trajectories when the outbreak was seeded in Dhaka (Fig. 3A, Supplementary Fig. 4). The mobility matrix generated from Meta, meanwhile, produced a delayed outbreak after similar initial growth in the first ~225 days. Meta’s epidemic took off in the seed city Dhaka earlier than the outbreaks generated from the other mobility sources, but the outbreak then took a while to reach all other districts, contrasting the other mobility sources’ more synchronously timed outbreaks (Fig. 3B, Supplementary Fig. 11).

When the outbreak was seeded in Chittagong, the gravity model predicted a slightly delayed outbreak compared to the three mobile phone operators (average outbreak peak at day 331 for the gravity model vs. day 313-315 for the operators, for ${R}_{0}=1.3$), while Meta’s outbreak was most delayed in timing (Fig. 3A). The gravity model, Meta, and Operator 1 generated early initial outbreaks in the seed city of Chittagong and delayed outbreak timing for all other districts (Fig. 3B, Supplementary Fig. 11).

**Fig. 4:**
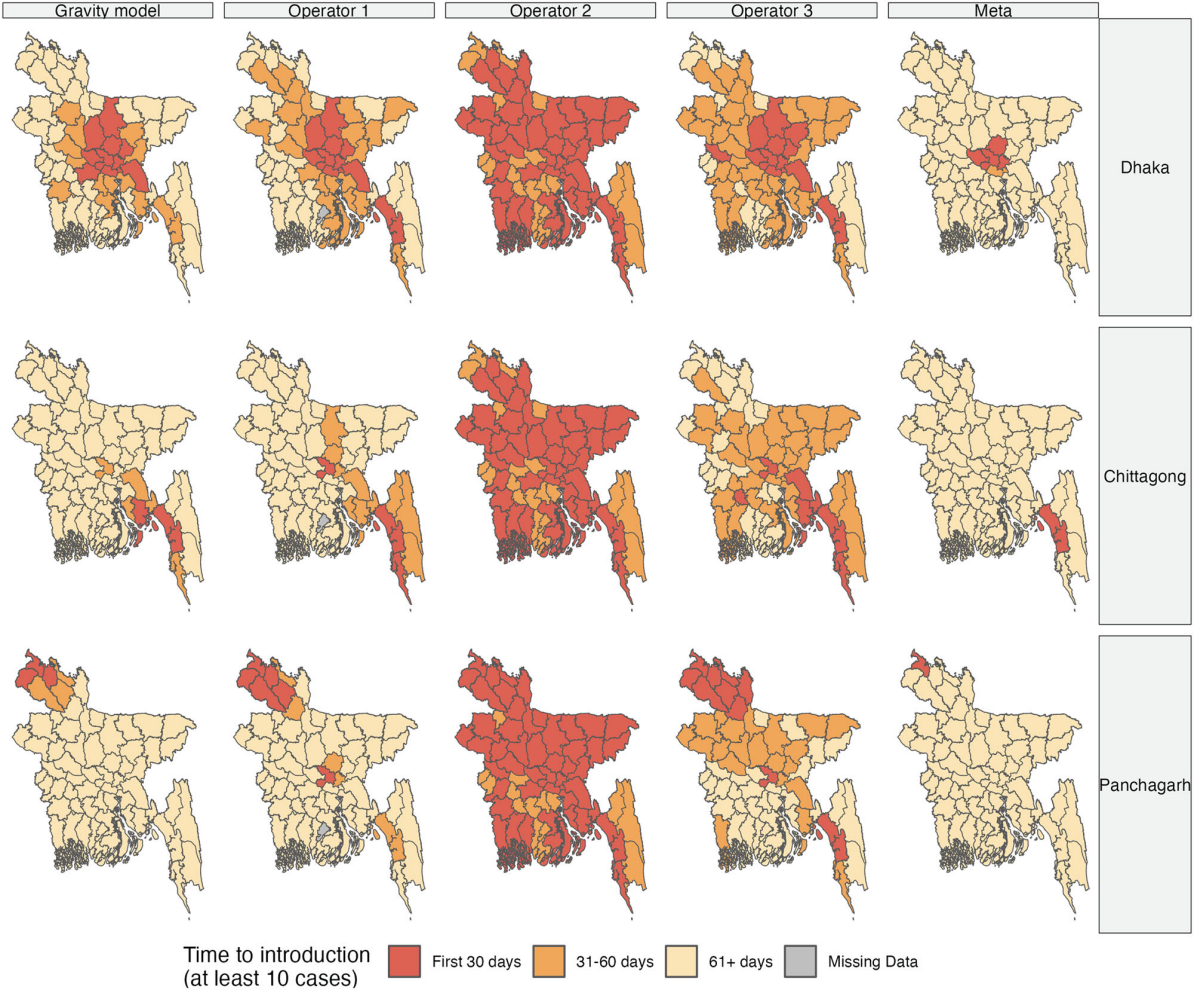
Spatial variation in outbreak timing by seed city and mobility data source. Time to outbreak introduction, defined as the day the district had at least 10 symptomatic cases, across districts by matrix mobility source and seed city.

The outbreaks from the three Operators and the gravity model were more differentiated when the outbreak was seeded in Panchagarh, which is a smaller and more geographically isolated city. This divergence was driven by different travel rates to Dhaka across the mobility sources, with the gravity model and Meta predicting slower outbreak introductions to Dhaka compared to the other sources (Fig. 4).In this scenario, however, Meta’s data produced a total incidence curve more similar to that of the other mobility sources because the outbreaks generated from the other sources also took a while to reach Dhaka, which has a crucial role in country-wide outbreak dynamics due to its connectedness to all other districts in each dataset. Meta’s delayed timing across districts therefore better matched the timing across districts predicted by the other mobility sources (Supplementary Fig. 11). When values of ${R}_{0}$ were increased from 1.2 to 2, the estimated outbreak trajectories showed similar patterns, though with faster outbreaks in all scenarios (Supplementary Fig. 4). Despite Operator 2’s relatively sparse upazila-level matrix, at the district level, across the different seed cities, it yielded outbreaks of similar total size compared to that of the other sources because it still predicted connectivity across all districts (Fig. 3A).

In terms of the heterogeneities in the spatial timing of district-level outbreaks, the gravity model predicted the timing of district-level outbreaks in non-seed cities to be in accordance with the district’s distance to the seed city (Fig. 3B). Models using the mobile phone operator data generally had earlier outbreaks than the gravity model, especially for more distant districts. This observation was most pronounced when the outbreak was seeded in Panchagarh; in this scenario the gravity model’s first outbreak to peak was the one in Panchagarh at 178 days and the last was Bandarban’s in southern Bangladesh on day 409. The district-level outbreaks generated from Operator 1’s mobility data had similar peak times across the different seed city simulations, centered at around 315 days, but more differences in timing emerged when the outbreak was seeded in Panchagarh, reflecting the city’s lower travel connectivity to other places compared to the seed cities of Dhaka and Chittagong. Meta’s outbreaks across districts were staggered over time, reflecting the data source’s lower levels of mixing between districts.

**Fig. 5:**
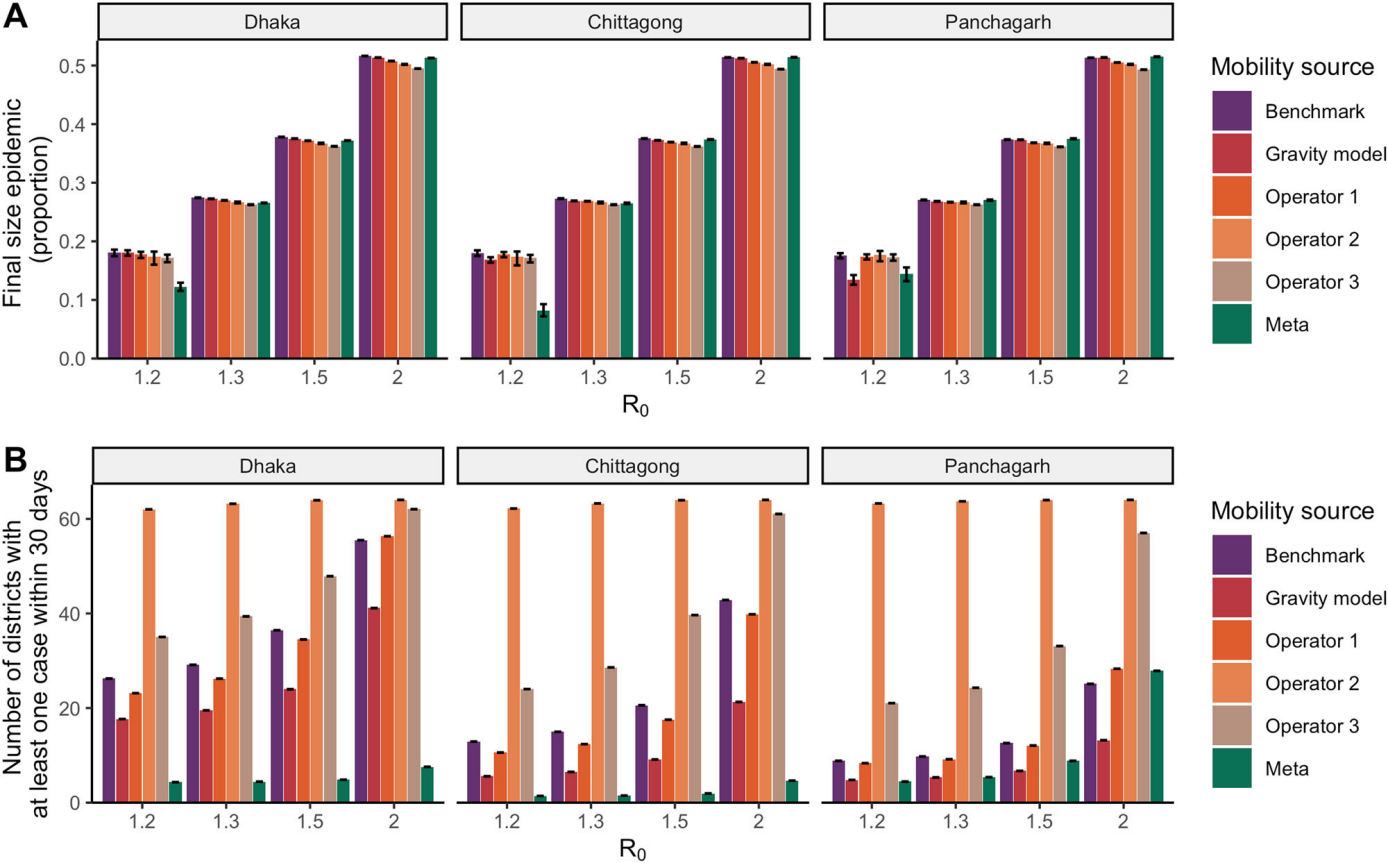
Differences in outbreak dynamics by seed city, mobility data source, and reproduction number. Final size of the epidemic (defined as the proportion of the cumulative number of symptomatic infectious individuals out of Bangladesh’s total population) by seed city and mobility source. 95% uncertainty bounds across 1000 simulations shown in error bars (**A**). Number of districts with at least one case within the first 30 days by seed city and mobility source. 95% uncertainty bounds across 1000 simulations shown in error bars (**B**).

Simulations with all mobility sources produced similar final outbreak size compared to the benchmark model (Fig. 3A, Fig. 5A). However, there was substantial heterogeneity in the timing of the outbreak spread, compared to the benchmark model (Fig. 3B). Simulations from Meta, Operator 1 and Operator 2 diverged the most from the benchmark; simulations using Meta generally had a slower spread, while simulations using Operator 2 suggested a much more rapid spread.

Differences in travel patterns across the mobility sources also affected the early spatial spread of the outbreak, where outbreak introduction was defined as the day a district reached at least 10 symptomatic infectious cases. For Operator 2 the high synchronicity in outbreak timing across districts (peak times ranging from 309 to 314 days) was due to the higher travel between places predicted by this data source, which resulted in more rapid spread across all districts relative to other mobility sources (Fig. 4). Operator 3 also had higher overall mobility than Operator 1, which similarly explained its faster spatial spread like Operator 2’s (Fig. 4). The early spatial spread of outbreaks using Meta’s data, by contrast, was limited to the districts neighboring each seed city due to the district-level matrix sparsity.

Due to the large outbreaks generated under all scenarios, the final epidemic size, defined as the cumulative incidence of symptomatic infections as a proportion of Bangladesh’s total population, was similar across mobility sources and within each ${R}_{0}$ and seed city scenario (Fig. 5A). In contrast with the similarity in final epidemic sizes across mobility sources, the early spatial spread of the outbreak varied more across districts (Fig. 5B). Across all seed cities and ${R}_{0}$ values, Operator 2’s data resulted in the highest number of districts with at least one case in the first 30 days of the outbreak; at ${R}_{0}$ values of 1.3 or higher, the outbreak spread to almost all 64 districts in the first 30 days with Operator 2’s data. By contrast, Meta’s data produced a much slower initial spread, ranging from 4 to 8 districts having at least one case within 30 days across ${R}_{0}$ values when seeded in Dhaka, 1 to 5 districts when seeded in Chittagong, and 4 to 28 districts when seeded in Panchagarh. Meta’s data lacked connections in many rural districts, such as those around Panchagarh. Because of the binomial sampling that accounted for few or no subscribers, mobility could be overestimated for some districts, resulting in the faster initial predicted spread when seeded in Panchagarh. Differences between Operators 2 and 3 for this metric, meanwhile, diminished at higher ${R}_{0}$ values of 1.5 and 2. In general, with higher values of ${R}_{0}$, the differences in the timing of the peak are less pronounced across simulations as the high transmissibility of the pathogen leads to rapid spread even for mobility sources with lower volumes of travel. In other words, when transmission rates are sufficiently high, the pathogen spreads even with relatively low connectivity across districts.

While the outbreak trajectories in terms of country-wide incidence were relatively similar across mobility sources in the district-level simulations, significant differences emerged with the upazila-level simulations (Supplementary Fig. 12). Using Operator 2 data, some or all outbreaks died out for scenarios where ${R}_{0}$ was 1.5 or lower due to the upazila-level sparsity of Operator 2’s mobility matrix. Across all scenarios, there was clearer divergence between each operator and the gravity model trajectories in terms of final size, peak size, and timing. The outbreak trajectories of Operators 1 and 3 become more similar only at higher ${R}_{0}$ values of 1.5 and 2.

**Fig. 6:**
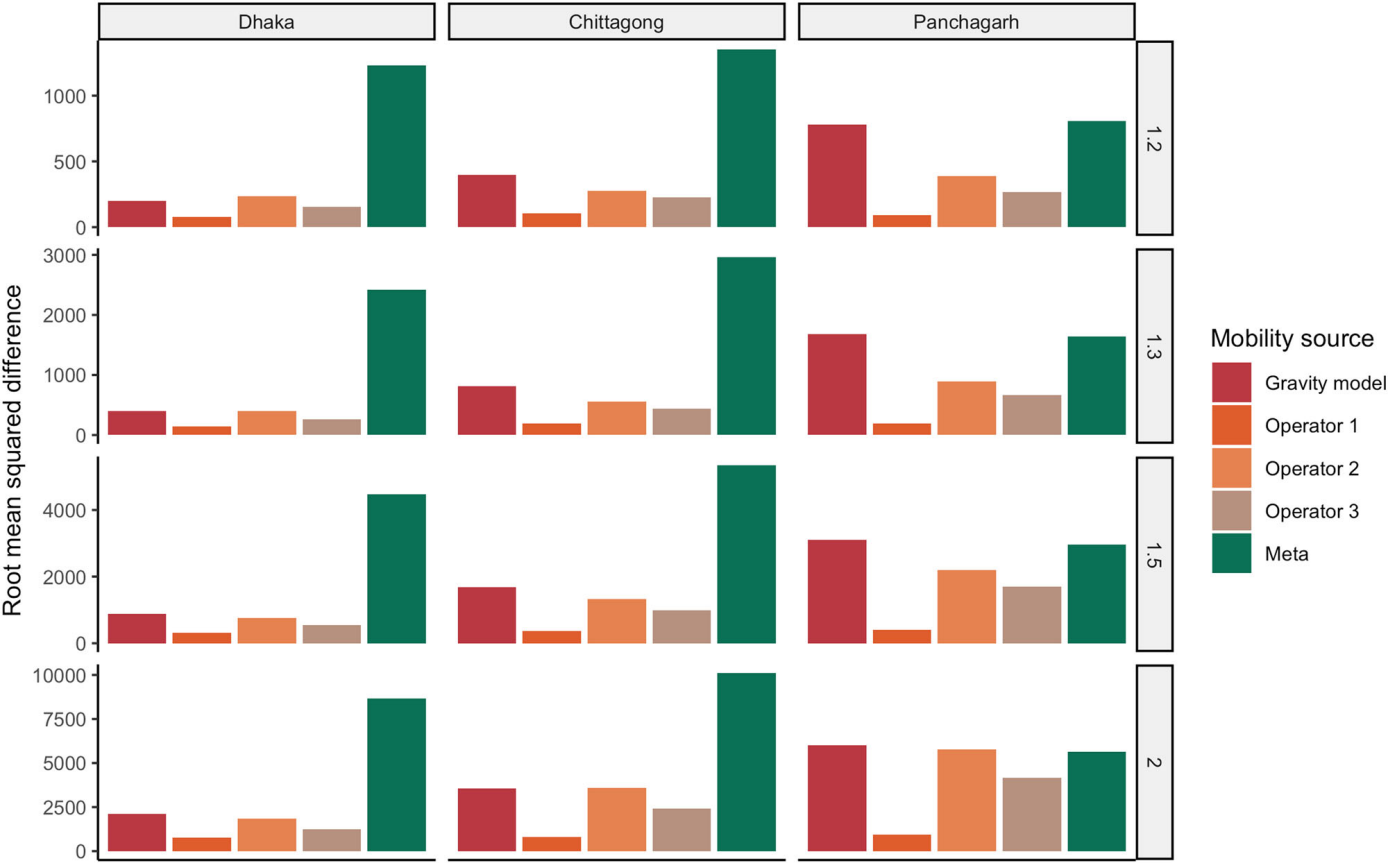
Difference between the simulated incidence for each mobility source and the combined dataset from all three operators (representing greatest population coverage). The difference is calculated as the square root of the mean squared difference across all time points and all districts. Differences are shown by seed city and ${R}_{0}$ values. Note that that y-axis scaling is different for each row since epidemic sizes, and therefore magnitude of differences, vary by ${R}_{0}$.

We compared the root mean squared difference from the benchmark model (Fig. 6), and found that across all values of ${R}_{0}$ and choice of seed city, Operator 1 had the smallest difference from the benchmark. Interestingly, simulations using data from Meta had the largest differences when the seed cities were Dhaka and Chittagong, suggesting that in many situations using a gravity model may provide more reliable estimates. In general, the gravity model performed well (comparable to simulations using data from Operator 2 and Operator 3) when the seed cities were large (Dhaka and Chittagong).

## Discussion

Given the importance of population level mobility on the transmission dynamics of infectious disease outbreaks, accurate measurements of mobility are critical for accurate modeling of outbreaks and for designing effective interventions. We demonstrate the significant differences in travel patterns, geographic coverage and volume of travel estimated from different sources of digital data from mobile phones and social media. This analysis sheds light on a methodological aspect of CDR analysis that has been underexplored – the bias that results from differences in operators’ geographic representativeness. Using data from three mobile phone operators in Bangladesh, we found that different data sources implied very different mobility patterns, including different levels of connectedness between locations and different overall levels of mobility. When compared with the benchmark model using the combined data from all three operators, we find several important results for guiding practitioners: 1) as expected datasets with greater population coverage performed better than simulations with more sparse data; 2) in many instances, the gravity model may be a better choice than using mobility sources with very low population coverage such as Meta; and 3) where an outbreak starts, the transmissibility of the pathogen, and the outcomes of interest, for example, peak incidence or timing of spatial spread, determines the magnitude of deviation from the benchmark.

The differences in the mobility sources had substantial impact on the estimated spread pattern of simulated epidemics, including whether the epidemics spread across the country, how fast they spread, the geographic pattern of spread, and the overall impact. These effects varied along the three dimensions of the simulated epidemics: (1) the city where the outbreak was seeded, (2) the spatial unit of analysis, and (3) the transmissibility of the pathogen. Simulated epidemics initialized in the large and centrally connected capital Dhaka, run at the district-level, and parameterized using higher ${R}_{0}$ values, exhibited relatively limited differences between the outbreak trajectories predicted by the different mobility sources. In contrast, initiation in less-connected locations, spread at finer geographical resolution, and lower ${R}_{0}$ values, which are all real-world considerations for epidemic models, accentuated differences.

For example, the district-level simulation results using Operator 2’s data had similar final epidemic size and overall outbreak timing to those of the gravity model and Operators 1 and 3. However, at the upazila level, Operator 2 mobility did not always produce country-wide epidemics, and the outbreak trajectories were very different between models, particularly at lower values of ${R}_{0}$. In a highly connected population, with a highly transmissible pathogen, and with minimal interventions, more sparse or biased datasets may have limited impact. However, with real heterogeneities in outbreak introductions, local scale spread, and lower transmissibility (which may even apply to high ${R}_{0}$ pathogens due to immunity or mitigation measures), these differences become much more important.

While simulations using the gravity model and each operator were similar in the conditions that produced large and fast epidemics, the simulations based on Meta’s mobility data differed even under these conditions. The sparsity of the district-level mobility matrix from Meta reflects the low uptake of smartphones and low usage of Meta apps in Bangladesh currently. These differences led to outbreaks across districts that were delayed and asynchronous in timing, resulting in comparable final epidemic sizes but divergent spatiotemporal dynamics compared to the other mobility data sources. The limitations of the mobility patterns captured by Meta are an important point of consideration. While these data offer immense promise, their coverage in low-resource settings, where they could potentially be most useful, may still be too low to use even for the simplest large scale metapopulation models.

Although we focus here on mobile phone operators in Bangladesh, our results are important to consider for any infectious disease modeling and prediction efforts using these novel sources of human mobility data. Our results suggest that the geographic representativeness of the data source, the spatial resolution of data available, the starting location of the outbreak, and the transmissibility of the pathogen are all important considerations when choosing a mobility data source for modeling and forecasting.

Our analysis has some important limitations and we make several simplifying assumptions in this analysis. While our model addresses geographic heterogeneity explicitly by modeling spread by mobility between locations (districts or upazilas), it assumes homogeneous mixing within those locations, an over-simplification of the complex local-scale transmission dynamics. CDRs do not capture many aspects relevant to transmission, such as transmission chains within upazilas, behaviors practiced while traveling (e.g. mask wearing, traveling more during non-peak hours, quarantining upon arrival at one’s destination), or type of travel (e.g. public transportation vs. private vehicles). We also assume that symptomatic individuals do not travel, which may underestimate the peak incidence and the introduction times to different locations. Finally, we do not consider variation in local conditions, such as healthcare infrastructure, social and cultural practices, local political environment, climate, etc., that can impact the spread of infectious diseases and lead to spatial heterogeneities in disease dynamics. These considerations may be important when simulating an actual epidemic, whereas our analysis was focused on exploring bias and therefore did not model a particular pathogen or past outbreak.

CDR and digital trace data have additional limitations to consider. First, they are often very difficult or impossible to attain, even from a single provider. They are also subject to biases due to heterogeneities in phone ownership and usage patterns. People who use cell phones on average likely travel more than the general population based on their demographic characteristics, like age, gender, and occupation^19,20^. Children in particular are typically not captured with CDR or digital trace data^37^, but are an important demographic group for disease transmission. In the context of the mobility matrices estimated here, the proportion of the population estimated to stay put in a geographic area versus travel to other geographic areas is likely higher in the general population than what is estimated using CDR data. Additionally, novel big data sources on human behavior, such as CDR and digital trace data, are generated using computer algorithms that may introduce more uncertainty and bias compared to traditional methods of data collection^38^.

Finally, a key limitation of this study is the lack of ground-truth data on population mobility in Bangladesh. While we use a benchmark model, comprising of data from all three operators, to evaluate the performance of each mobility source, the benchmark mobility is also plagued by the same limitations as described above, although likely to a lesser degree as it represents a significant portion of the country’s population. To address this important methodological challenge, future work should be focused on developing methods for collecting population-representative high-resolution data on movement patterns, for example, through frequent nationally representative surveys, and on generating metadata for CDR data that includes demographic characteristics that could be used to correct for sampling biases.

In light of these findings, sensitivity analyses may help capture uncertainty in a mobility data source with limited geographic representativeness. These analyses could vary the proportion of people assumed to stay in their origin district and could increase the connectivity implied by the dataset (*i.e*. reduce matrix sparsity by making assumptions for location pairs with no trips). Future work could also use other data sources, such as travel surveys and questionnaires, to estimate the proportion of people that stay put on a given day and adjust the matrices to reflect travel patterns that are more representative of those of the general population. Another research priority involves continuing to develop and compare metrics that can be calculated from alternative data sources, such as travel surveys and census data, and CDRs^37,39,40^.

In an increasingly interconnected world, epidemic preparedness and response must account for how populations are interconnected. Here, we demonstrate that even with what might be considered ideal datasets of phone level mobility for large sectors of the population of Bangladesh and a relatively simple simulated epidemic, expected dynamics are highly variable depending on which mobility data are available. Modeling analyses that are reliant on a single mobile phone operator, unless the operator captures a significant proportion of the underlying population base, likely reflect the biases of the operator’s geographic footprint. Digital data sources provide unprecedented opportunities for estimating critical population mobility fluxes with real-time data, yet understanding and accounting for their biases remains an important area of research. This work contributes to the debate on the generalizability of models built on these types of human behavioral data that are increasingly being used in public health, and highlights the potential pitfalls of using these data for providing mechanistic insights into disease transmission.

## Supplementary information

Supplementary Information

Reporting Summary

## Data Availability

The CDR data that support the findings of this study are available on request from a2i. a2i anonymized the CDR data to ensure the security of the privacy and sensitivity of the data. The Meta datasets are available to researchers through Meta upon request from Meta’s Data for Good program.

## Code availability

Code for our analyses is available on Github at https://github.com/taylor-chin/bgd-mobility-release.
