# Supplementary material for: Bias in mobility datasets drives divergence in modeled outbreak dynamics
Source: Commun Med (Lond). 2025 Jan 7;5:8. doi: 10.1038/s43856-024-00714-5 (PMC11706981; doi:10.1038/s43856-024-00714-5)
Supplement: Supplementary file 1 — Supplementary Information [file 43856_2024_714_MOESM1_ESM.pdf]

Supplementary Information

Supplementary Methods

Missing data
The raw data received from the operators included the average number of subscribers traveling from upazila *j* to upazila *i* on a weekday or weekend day in 2020 for Operators 1-3. If an operator reported any travel from a given upazila, *a,* but no travel between upazila *a* and upazila *b*, we assumed no subscribers traveled between these upazilas (*i.e.* mobility estimate of zero) since a lack of cell towers in the upazilas was not an explanation for the lack of reported travel. By contrast, we counted upazilas without any travel data–either travel to or from–as having true missing values, possibly representing upazilas with a lack of cell phone tower coverage for that operator. These true missing upazilas represent upazilas for which all row and column elements in **R** are zero. In Operator 1's and Operator 2's data, approximately 3% out of 544 total upazilas were missing. In Operator 3's data approximately 6% of upazilas were missing.

Operators 1 and 3 also had missing data in terms of travel to and from a subset of Dhaka upazilas. Given the high urbanicity of Dhaka, we assumed that a lack of cell tower coverage was not driving this missingness, but instead, possibly a data processing and/or reporting issue. We imputed estimates for the travel to and from the missing Dhaka upazilas using a set of assumptions and data from Operator 1's 2017 data. We first excluded the dates June 25-27, 2017 and September 1-3, 2017 from the 2017 data since these represent days where travel was atypical due to the Eid holidays. To test the validity of using 2017 data to impute missing 2020 data, we calculated the correlation between the proportions of trips from each Dhaka upazila to all non-Dhaka districts in 2017 and 2020 using Operator 1's data (**Supplementary Fig. 13**). We subset the Dhaka upazilas to the ones that were non-missing in both the 2017 and 2020 data for this comparison. Given the high correlation between the proportions (R = 0.97), we assumed the years 2017 and 2020 had similar average travel patterns from Dhaka. In the absence of available 2017 data for Operator 3, we used the proportions of trips from Operator 1's 2017 data to impute Operator 3's missing Dhaka upazilas. As performed for the 2020 data, we generated the mobility matrix, **T**, with elements t_{ij}, representing the average daily number of trips taken by subscribers between pairs of upazilas in Operator 1's 2017 data. We refer to the 2020 mobility matrices with imputed 2017 values for missing Dhaka upazilas as **Q** with elements q_{ij}.

To impute the number of trips from an upazila *a,* into a missing Dhaka upazila, *b,* we first took the corresponding number of trips from upazila *a* to upazila *b* from Operator 1's 2017 data. Since 2020 absolute travel volumes differed from those of 2017 because of a secular annual increase in travel volume and because of disruptions in travel volume during the pandemic, we used the upazilas in Operator 1's data in both 2017 and 2020 and calculated a multiplier, defined as the 2020 travel volume divided by the corresponding 2017 travel volume for each pairwise upazila combination. Lastly, we multiplied the 2017 estimated number of trips by the median multiplier estimated across all pairwise upazilas, which was approximately 1.33.

To impute the number of trips from a missing Dhaka upazila to all other upazilas and number of subscribers who stay put in that upazila, we used the corresponding number of trips from Operator 1's 2017 data. Multiplying these values by the median multiplier was not necessary because only the proportions from these columns, and not the absolute values, were used to construct the matrices, as described below. In summary, elements of **Q** were calculated based on elements of **T** according to the rules:

$$q_{ij} = \begin{cases} t_{ij} * 1.33 & i \in z \; for \; all \; i \neq j \\ t_{ij} & j \in z \\ r_{ij} & i \notin z \; and \; j \notin z \end{cases}$$

where z is the set of missing Dhaka upazilas.

Parameters	Weekday Model	Weekend Model
$\mathrm{Log}(k)$	-15.80	-16.84
	[-16.74; -14.86]	[-17.83; -15.86]
α	1.05	1.11
	[1.01; 1.10]	[1.06; 1.15]
β	1.04	1.08
	[1.00; 1.08]	[1.04; 1.13]
γ	-1.96	-2.05
	[-2.00; -1.91]	[-2.10; -2.00]
AIC	51583.84	50972.48
BIC	51615.35	51003.99
Log Likelihood	-25786.92	-25481.24
Num. obs.	4032	4032

Supplementary Table 1. Gravity model parameters (and 95% confidence intervals) estimated by fitting negative binomial regression models, separately for weekdays and weekends, to the combined CDR data for all three operators. The dependent variable was the count of trips between locations; the independent variables were populations in the origin and destination locations (in log scale) and the distance between locations (in log scale). The estimated coefficients correspond to the gravity model parameters in Equation (1) of the manuscript.

**Spatial level of analysis**	**District**	**Upazila**
Mobility sources	CDR Operators 1-3, Meta Data for Good, Gravity model	CDR Operators 1-3, Gravity model
R_0 values	1.2, 1.3, 1.5, 2	1.2, 1.3, 1.5, 2
Seed cities	Dhaka, Chittagong, Panchagarh	Dhaka, Chittagong, Panchagarh
Initialization	500 people in latent compartment of seed city's district	100 people each in latent compartment of the 5 most populous upazilas in seed city's district

Supplementary Table 2. Summary of initial conditions for metapopulation simulations run at the district and upazila level

Mobility matrix	Definition
**R**	Average number of subscribers traveling from upazila *j* to upazila *i* on a weekday or weekend day in 2020 for Operators 1-3
**T**	Average number of subscribers traveling from upazila *j* to upazila *i* on a weekday or weekend day in 2017 from Operator 1
**Q**	Average number of subscribers traveling from upazila *j* to upazila *i* on a weekday or weekend day in 2020 for Operators 1-3, where values for Operator 1 and 3's missing Dhaka upazilas were imputed using **T (see Supplementary Methods)**

**B**	The probability of travel from upazila *j* to upazila *i* on a weekday or weekend estimated by drawing from a beta distribution fit to each element in **Q**
**H**	Normalized matrix **B**, where matrix elements were divided by their corresponding column sums, such that each column sums to 1
**K**	Average number of people in the population traveling from upazila *j* to upazila *i* on a weekday or weekend day in 2020 for Operator 1-3
**S**	Average number of people in the population traveling from upazila *j* to upazila *i* on a weekday or weekend day in 2020 for Operator 1-3, where the matrix has been made symmetric to balance population inflows and outflows
**D**	Average number of people in the population traveling from district *j* to district *i* on a weekday or weekend day in 2020 for Operator 1-3
**M**	Either **S** for upazila-level simulations or aggregated to district level, **D**, for district-level simulations

Supplementary Table 3. Summary of matrices used to create final origin-destination mobility matrices

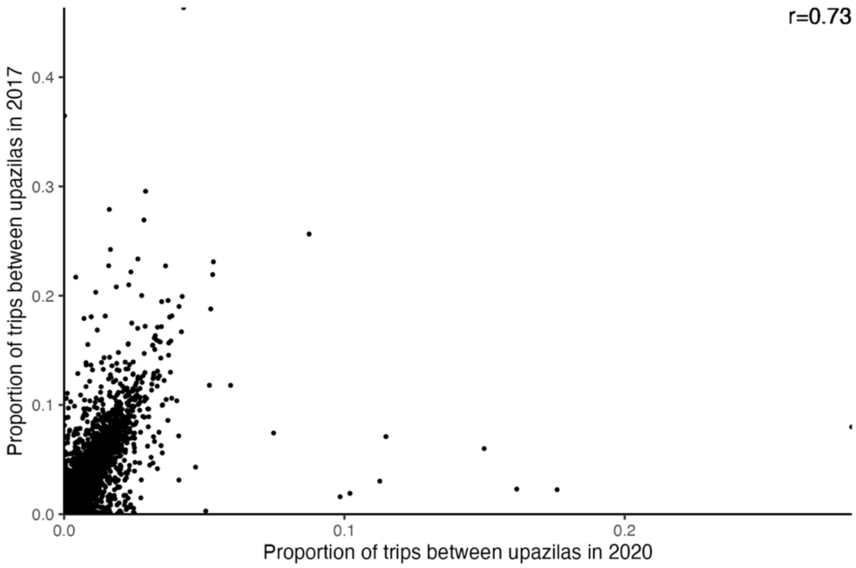

Supplementary Figure 1. The proportion of number of trips from an upazila to all other upazilas from Operator 1's 2017 and 2020 data. The correlation coefficient between the proportions is printed in the top right corner.

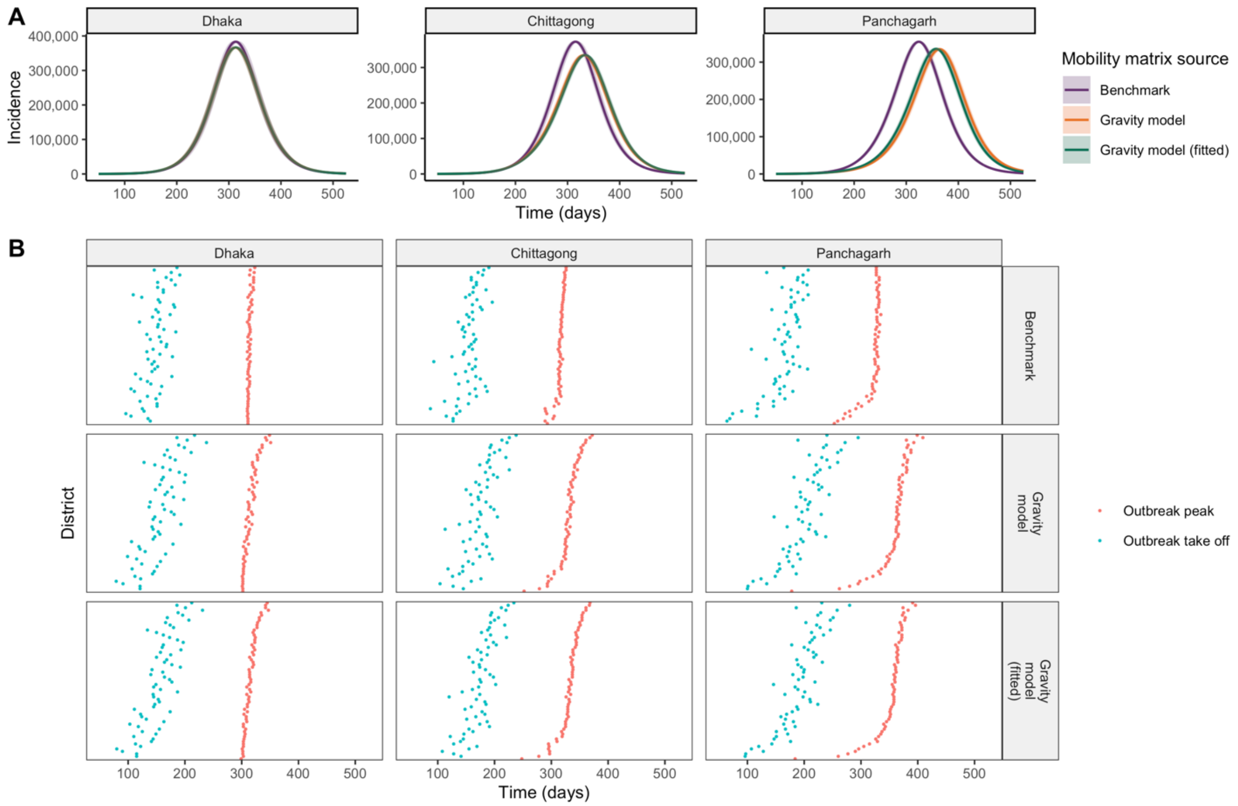

Supplementary Figure 2. Incidence of symptomatic, infectious individuals for all of Bangladesh over time by seed city for the benchmark model, the gravity model with parameters from literature, and the gravity model with parameters estimated by fitting a negative binomial regression model to combined CDR data from Operators 1, 2, and 3 (A). Time the outbreak peaks and time the outbreak takes off (≥50 cumulative symptomatic infected cases) across districts by seed city and mobility matrix source. The district order on the y-axis is ordered by distance to the seed city, with the closest district to the seed city on the bottom and the farthest district on the top of each plot (B). For all simulations, $R_0 = 1.3$.

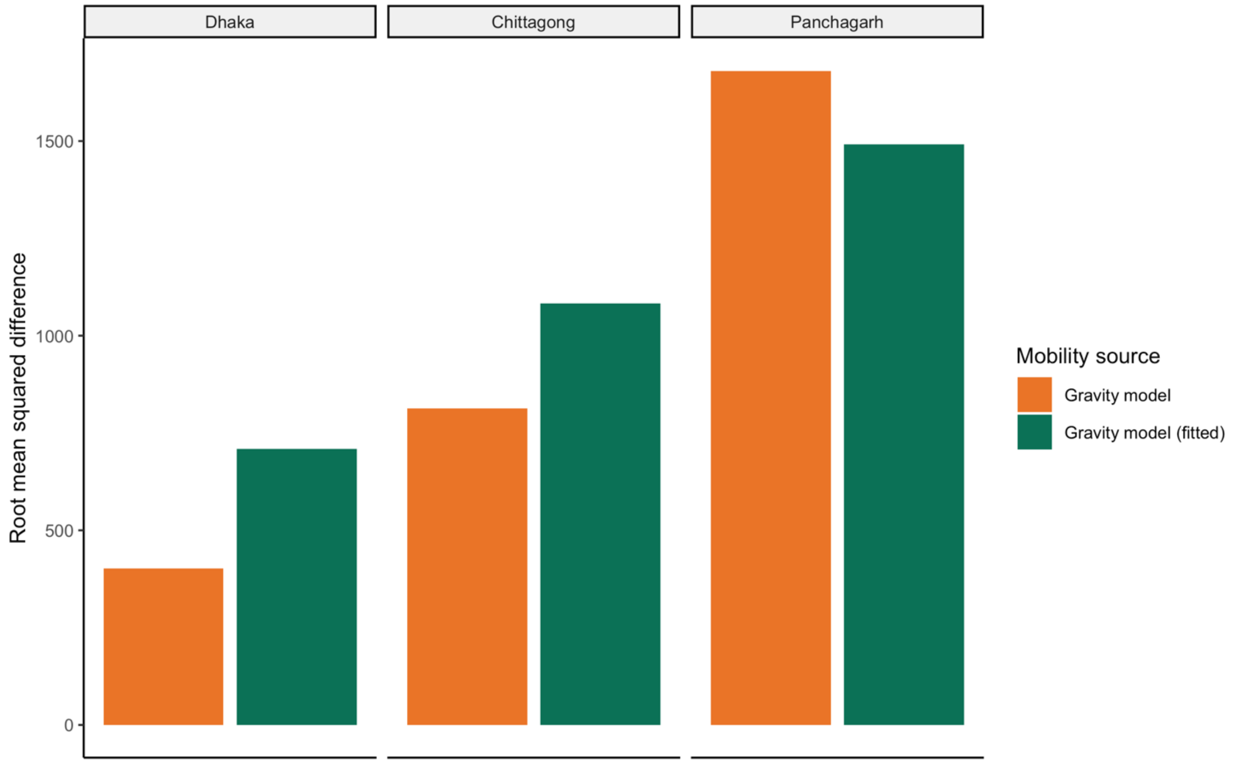

Supplementary Figure 3. Difference between the simulated incidence for the benchmark model and the gravity model with parameters from literature and the gravity model with parameters estimated by fitting a negative binomial regression model to combined CDR data from Operators 1, 2, and 3. The difference is calculated as the square root of the mean squared difference across all time points and all districts. Differences are shown by seed city. For all simulations, $R_0 = 1.3$.

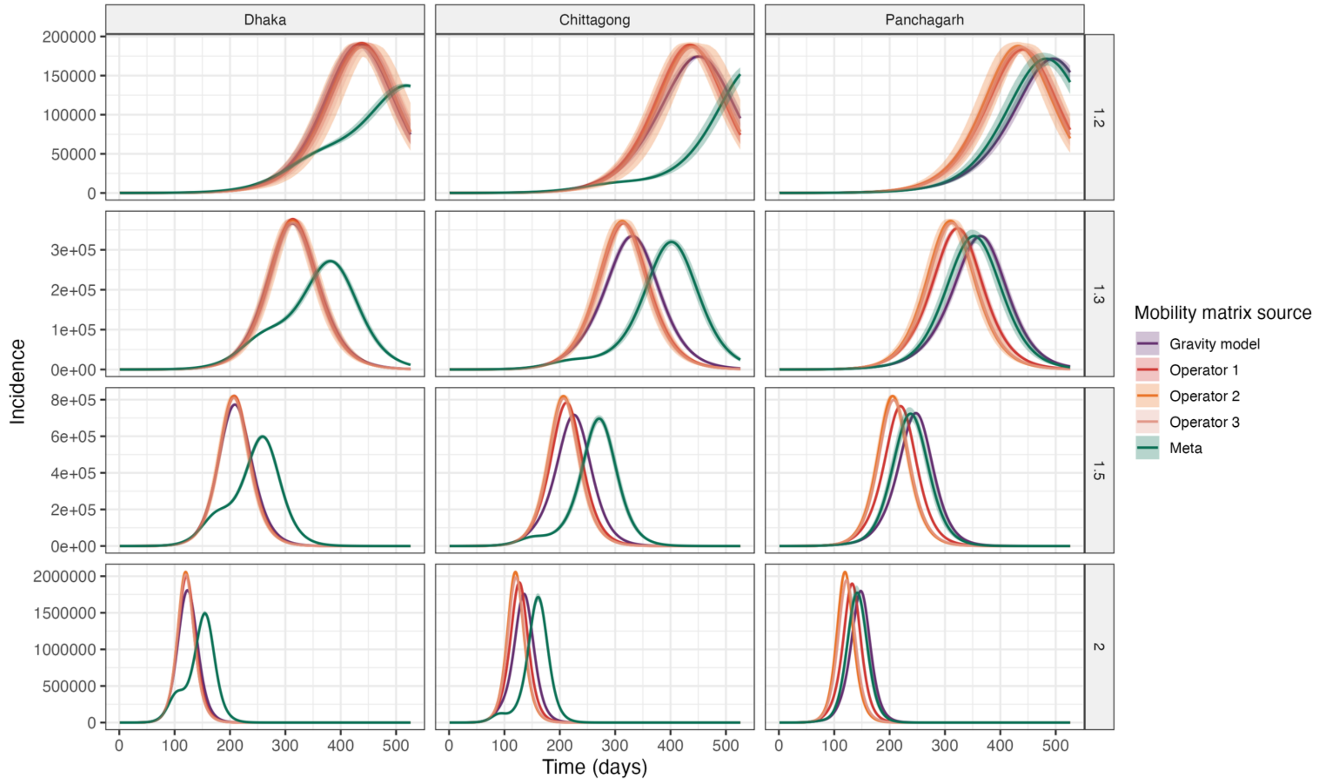

Supplementary Figure 4. District-level simulation results (total incidence for all of Bangladesh) for Operators 1-3, Meta, and the gravity model by seed city and R_0. Incidence refers to daily number of new symptomatic, infectious individuals. Simulations were seeded with 500 people in the latent compartment in each of the seed city's district.

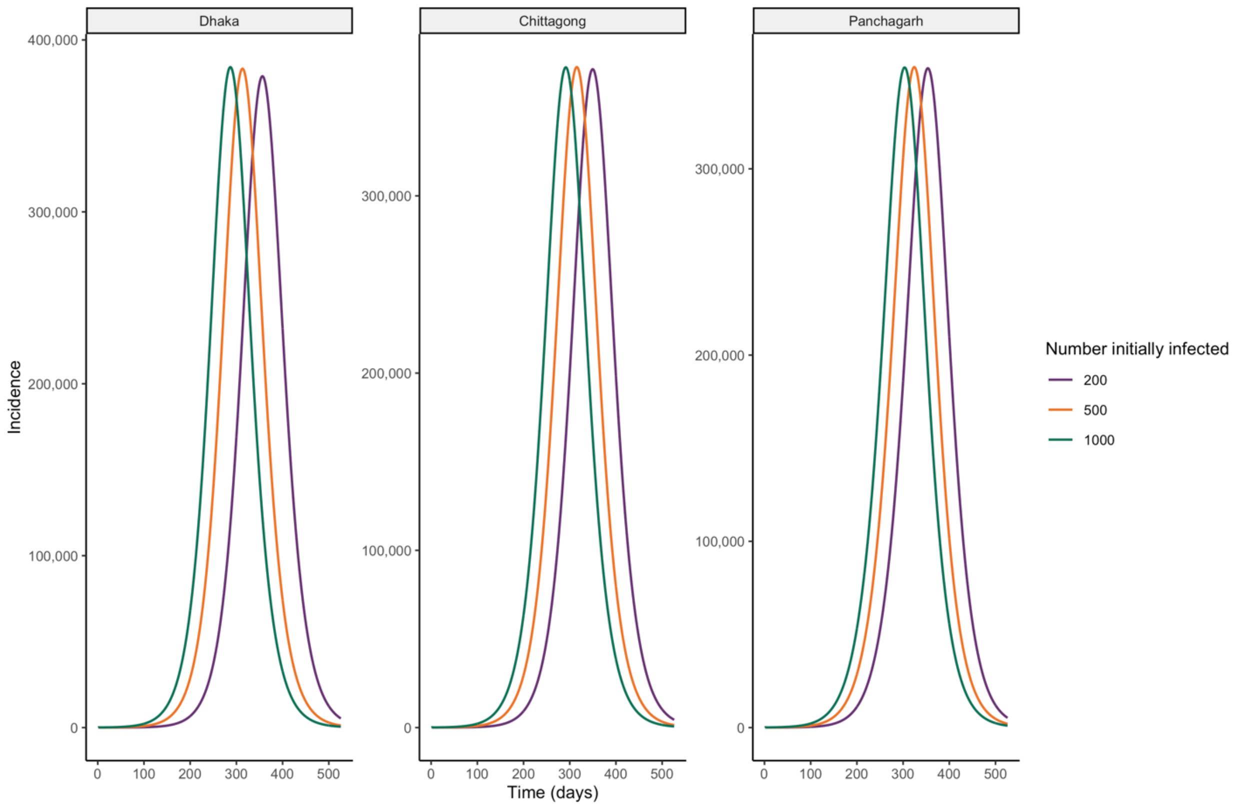

Supplementary Figure 5. District-level simulation results (total incidence for all of Bangladesh) for all three operators combined (benchmark model) by seed city and initial number infected. Incidence refers to the daily number of new symptomatic, infectious individuals. Simulations were seeded with 200, 500, and 1000 people in the latent compartment in each of the seed city's districts.

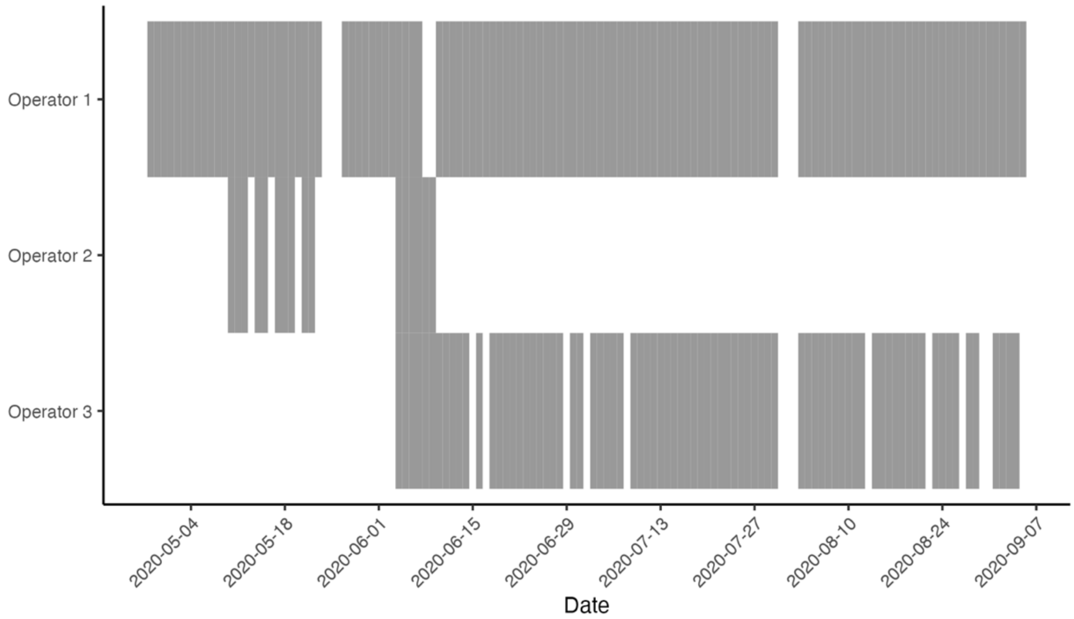

Supplementary Figure 6. Dates with available data for each CDR operator in 2020

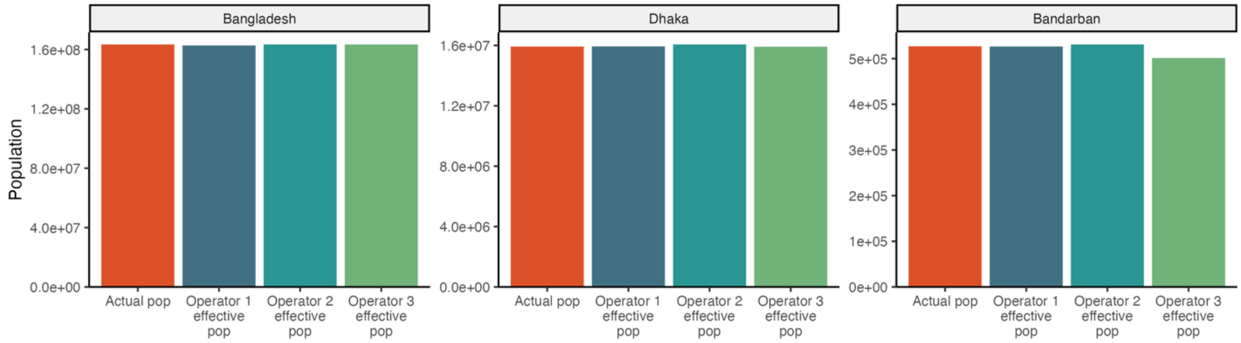

Supplementary Figure 7. Actual population size versus effective population sizes from Operators 1-3's matrices for Bangladesh, Dhaka, and Bandarban

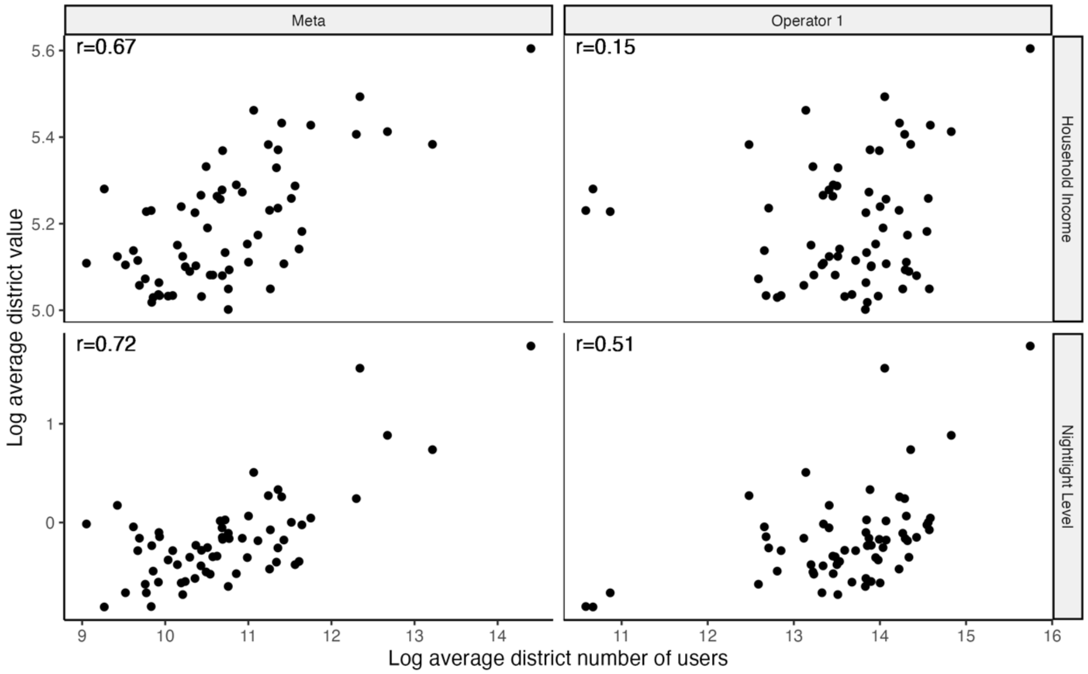

Supplementary Figure 8. Scatterplots of the log average district number of users (Meta users or Operator 1 subscribers) versus the log average district value of a demographic measure (household income level (USD) or nightlight level)

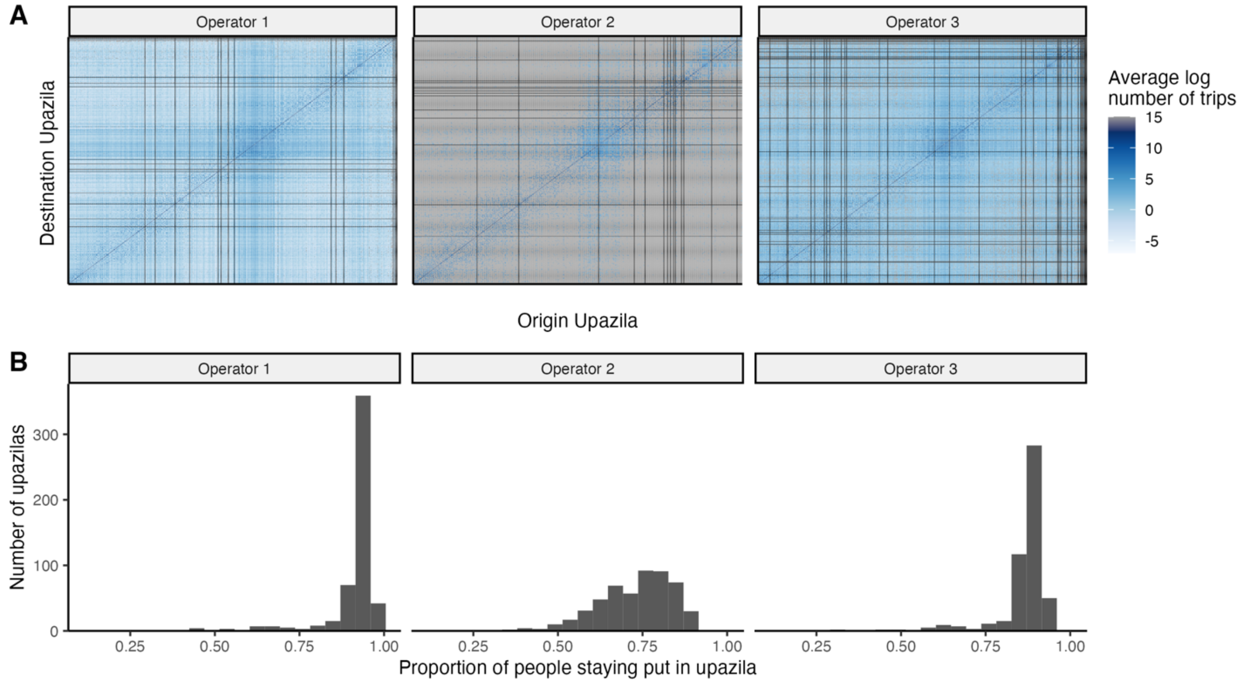

Supplementary Figure 9. Upazila-level origin-destination matrices, showing the daily average log number of trips taken by people in the population on weekdays in 2020 after excluding the Eid holidays. The matrices have been made symmetric, and upazilas have been ordered by latitude (A). Black horizontal and vertical lines represent true missing upazilas, or upazilas that lack data entirely in the operator's data. Grey matrix values represent a value of zero trips (*i.e.* undefined log number of trips) between upazila pairs. Histograms of the proportions of people staying put in an upazila out of the total number of people traveling from that upazila or staying put across all upazilas (B).

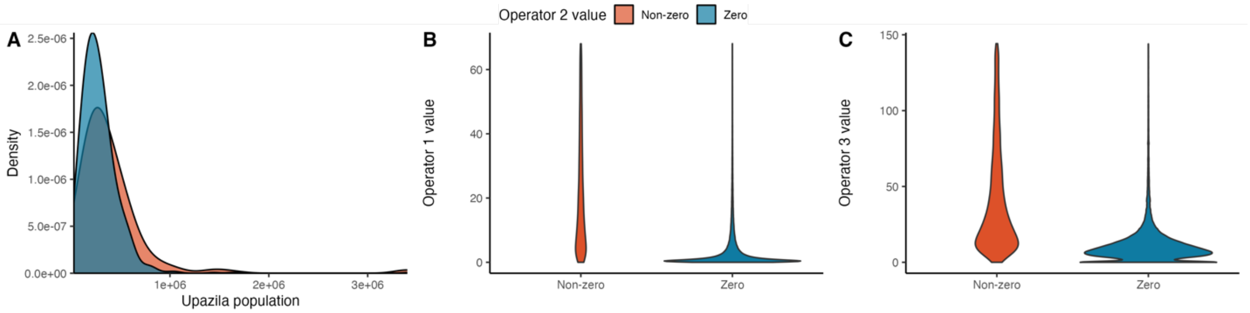

Supplementary Figure 10. Density plot of the populations of origin upazilas, separated by upazilas with zero vs. non-zero Operator 2 values for the daily average number of trips (A). The daily average number of trips on weekdays in 2020 (excluding Eid holidays) between upazilas in Operator 1's data for corresponding upazila pairs in Operator 2's data, separated by zero vs. non-zero Operator 2 values (B). The daily average number of trips on weekdays in 2020 (excluding Eid holidays) between upazilas in Operator 3's data for corresponding upazila pairs in Operator 2's data, separated by zero vs. non-zero Operator 2 values (B).

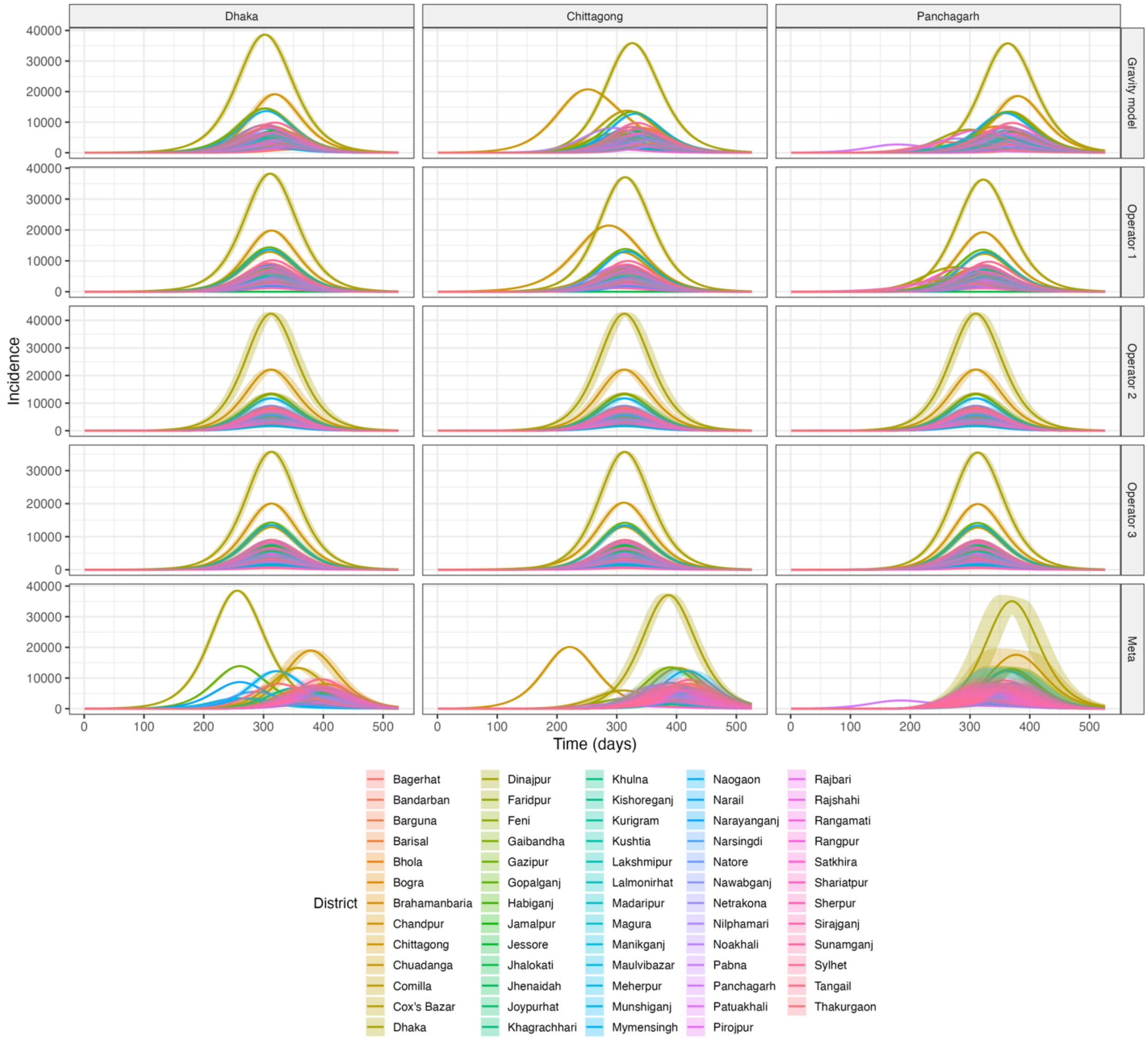

Supplementary Figure 11. District-level simulation results (district-level incidence) for Operators 1-3 and the null gravity model by seed city and $R_0 = 1.3$. Incidence refers to daily number of new symptomatic, infectious individuals. Simulations were seeded with 500 people in the latent compartment in each of the seed city's district.

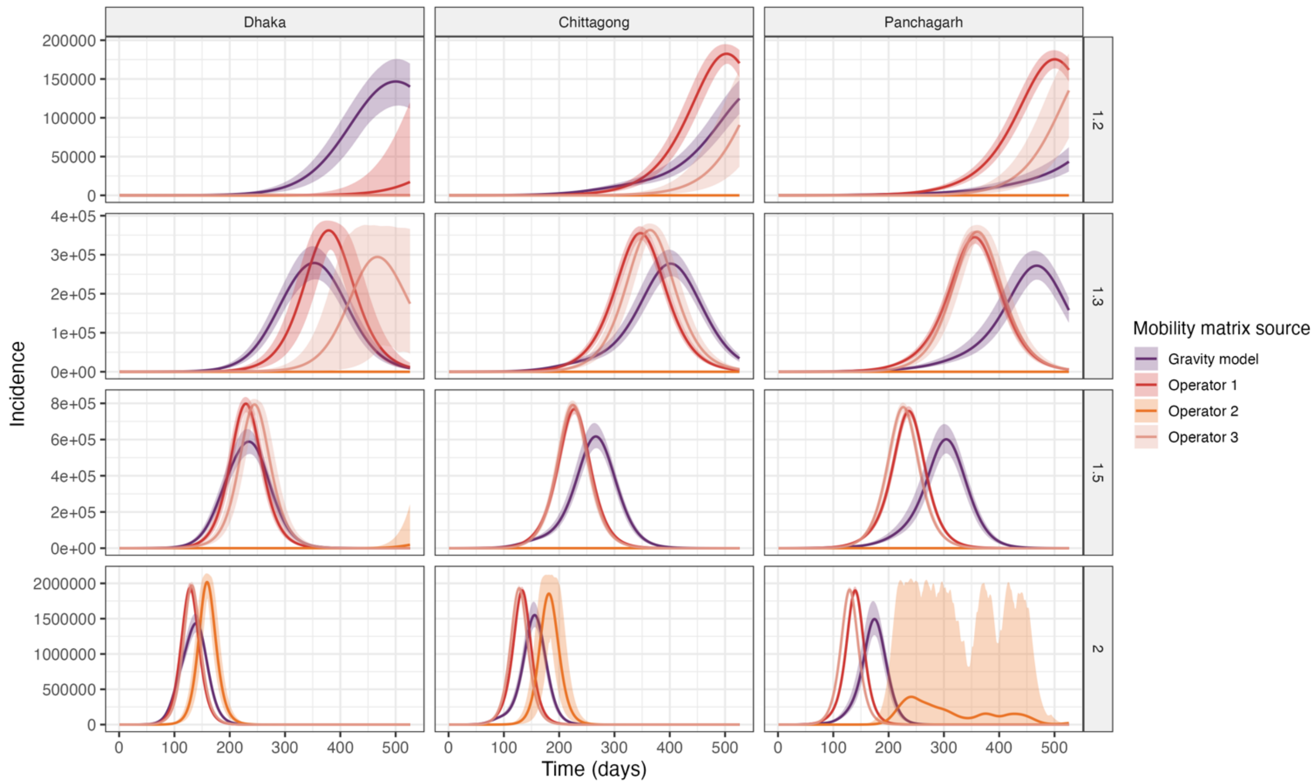

Supplementary Figure 12. Upazila-level simulation results (total incidence for all of Bangladesh) for Operators 1-3 and the null gravity model by seed city and R_0. Incidence refers to daily number of new symptomatic, infectious individuals. Simulations were seeded with 100 people in the latent compartment in each of the five most populous upazilas in the seed city.

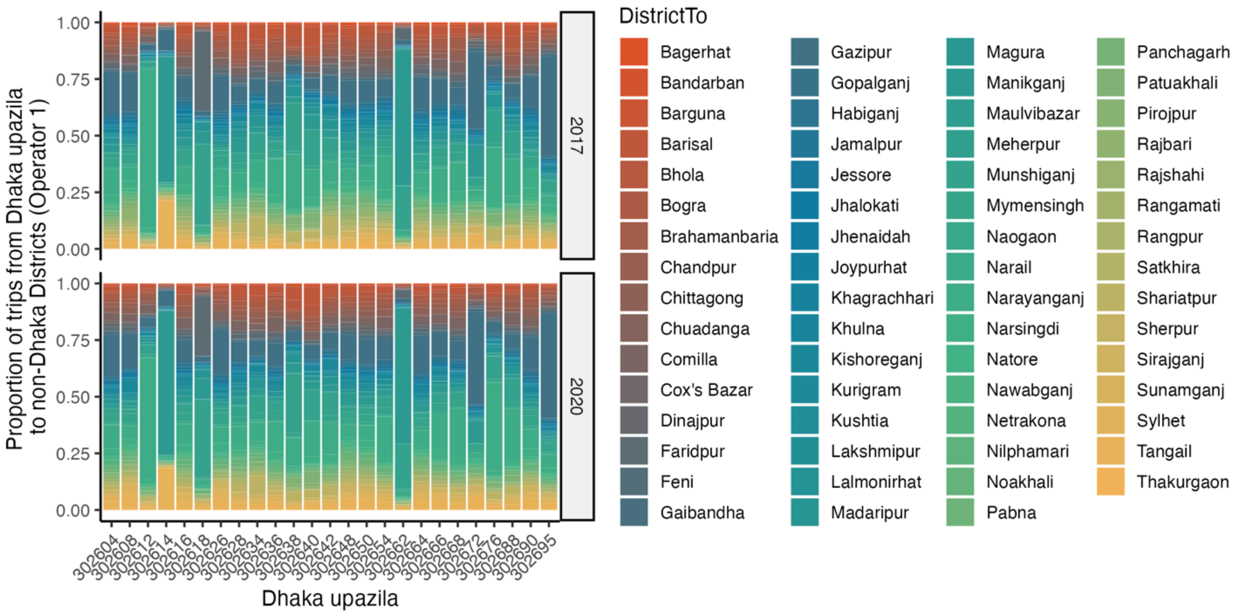

Supplementary Figure 13. The proportion of trips from individual Dhaka upazilas to all non-Dhaka districts from Operator 1's 2017 and 2020 data.
